# Tilting and Distortion in the Multiferroic Aurivillius Phase Bi_6_Ti_3_Fe_1.5_Mn_0.5_O_18_

**DOI:** 10.1021/acs.chemmater.4c00413

**Published:** 2024-05-29

**Authors:** Louise Colfer, Núria Bagués, Mohammad Noor-A-Alam, Michael Schmidt, Michael Nolan, David W. McComb, Lynette Keeney

**Affiliations:** † Tyndall National Institute, University College Cork, Lee Maltings Complex, Dyke Parade, Cork T12 R5CP, Ireland; ‡ Center for Electron Microscopy and Analysis, The Ohio State University, Columbus, Ohio 43212, United States; § Department of Materials Sciences and Engineering, The Ohio State University, Columbus, Ohio 43210, United States

## Abstract

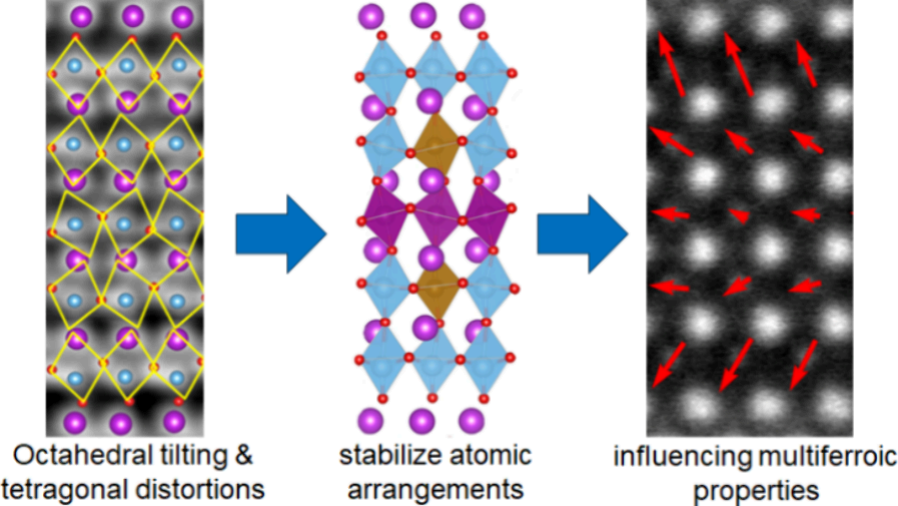

Aurivillius structured Bi_6_Ti_3_Fe_1.5_Mn_0.5_O_18_ (B6TFMO) has emerged as a rare room temperature multiferroic, exhibiting reversible magnetoelectric switching of ferroelectric domains under cycled magnetic fields. This layered oxide presents exceptional avenues for advancing data storage technologies owing to its distinctive ferroelectric and ferrimagnetic characteristics. Despite its immense potential, a comprehensive understanding of the underlying mechanisms driving multiferroic behavior remains elusive. Herein, we employ atomic resolution electron microscopy to elucidate the interplay of octahedral tilting and atomic-level structural distortions within B6TFMO, associating these phenomena with functional properties. Fundamental electronic features at varying bonding environments within this complex system are scrutinized using electron energy loss spectroscopy (EELS), revealing that the electronic nature of the Ti^4+^ cations within perovskite BO_6_ octahedra is influenced by position within the Aurivillius structure. Layer-by-layer EELS analysis shows an ascending crystal field splitting (Δ) trend from outer to center perovskite layers, with an average increase in Δ of 0.13 ± 0.06 eV. Density functional theory calculations, supported by atomic resolution polarization vector mapping of B-site cations, underscore the correlation between the evolving nature of Ti^4+^ cations, the extent of tetragonal distortion and ferroelectric behavior. Integrated differential phase contrast imaging unveils the position of light oxygen atoms in B6TFMO for the first time, exposing an escalating degree of octahedral tilting toward the center layers, which competes with the magnitude of BO_6_ tetragonal distortion. The observed octahedral tilting, influenced by B-site cation arrangement, is deemed crucial for juxtaposing magnetic cations and establishing long-range ferrimagnetic order in multiferroic B6TFMO.

## 1.0 Introduction

Data storage devices based on multiferroic materials, possessing simultaneous ferroelectric and magnetic (that is ferromagnetic or ferrimagnetic) memory states have the potential to transform future information storage,^1,2^ conceivably serving as unique multilevel memory elements in the quest for low-power neuromorphic memory systems.^3,4^ In practice, due to the fundamental opposing requirements of ferroelectricity (requiring empty *d* orbitals) and ferromagnetism/ferrimagnetism (requiring partially occupied *d* orbitals), single-phase multiferroic materials that display room temperature (RT) operation are exceptionally rare.^5^ Of the multiferroic materials that do exist, the material properties relevant for multistate storage are currently limited to weak polarization and coupling values, which has prevented the realization of such devices commercially.^1^ BiFeO_3_ is the most widely studied single phase multiferroic material^6^ but suffers from issues including the following: the magnetic order is primarily antiferromagnetic and it displays only a weak (∼2 emu/cm^3^) residual moment from a canted spin structure. Direct linear magnetoelectric coupling between polarization and magnetization in BiFeO_3_ is forbidden by preserved inversion symmetry and only higher-order magnetoelectric effects are possible with the assistance of large threshold magnetic fields (∼3 to 18 T).^7^ This is clearly incompatible with low-energy spintronics.^1,8^

Despite the fundamental conflict between ferroelectricity and ferromagnetism, innovative routes have been employed toward the design and discovery of truly single phase multiferroic materials, including a group of materials termed “geometrically driven multiferroics”, where ferroelectricity results from long-range dipole–dipole interactions and oxygen rotations within a magnetic framework.^9^ For example, improper ferroelectricity (*T*_c_ ∼ 1250 K) in YMnO_3_ is driven by polyhedral tilting of the MnO_5_ block and is compatible with the coexistence of antiferromagnetism (*T*_N_ ∼ 80 K).^10,11^ Even with substantial research on other hexagonal RMnO_3_ systems (where R = Sc, Y, In, Dy, and Lu),^10^ a rare earth manganite that demonstrates both ferroelectricity and ferromagnetism at room temperature is yet to be discovered.^5^

An attractive approach is to modify the chemistry within layered materials to combine multiple functionalities that tend not to coexist in simpler oxides into a single-phase complex oxide. Pitcher et al.^12^ employed compositionally controlled tilt engineering in a layered Ruddlesden–Popper system to impose the appropriate combination of octahedral tilts to simultaneously generate spontaneous magnetization and polarization at RT. Although ferroelectric switching was not demonstrated, it was shown that the magnetic structure of the (Ca_*y*_Sr_1–*y*_)_1.15_Tb_1.85_Fe_2_O_7_ system was directly affected by the chemically induced polar *c*^+^ tilt, with weak ferromagnetism produced by canting of the Fe^3+^ moments and the magnitude of the magnetoelectric coupling increasing with the magnitude of the *c*^+^ tilt.

In recent years, we have demonstrated that the fundamental contra-indications between ferroelectricity and ferromagnetism/ferrimagnetism^5^ can be circumvented by combining the chemistries required for multiferroicity through creating a new layered Aurivillius composition.^13−15^ Bismuth-based Aurivillius phase materials are naturally layered structures with a general formula (Bi_2_O_2_)(A_*m*–1_B_*m*_O_3*m*+1_), where dielectric bismuth oxide (Bi_2_O_2_)^2+^ fluorite-type layers are interleaved with *m* numbers of ferroelectric perovskite-type units. Aurivillius phases are considered “proper” ferroelectrics where ferroelectricity arises from polar distortions of the A-site (e.g., through stereochemical activity of the Bi^3+^ 6s^2^ lone pair electrons) and B-site (e.g., through second order Jahn–Teller distortions of Ti^4+^) cations. Tilts or rotations of the BO_6_ octahedra contribute to nonpolar structural distortions.^16^

The flexible and layered nature of this class of material permits engineering its composition to modify and enhance key properties, e.g., incorporating Co can alter the band gap.^17^ The (Bi_2_O_2_)^2+^ layers play a crucial role in charge compensation, contributing to reduced leakage currents and enhanced fatigue resistance characteristics compared to conventional perovskite materials. Consequently, Aurivillius phases, like SrBi_2_Ta_2_O_9_, SrBi_2_NbTaO_9_, and SrBi_4_Ta_4_O_15_, have found applications in high-speed, low-power, nonvolatile ferroelectric random access memory devices.^18,19^ The use of in-plane polarized Bi_5_Ti_3_FeO_15_ Aurivillius phase as a buffer layer in geometrically engineered BaTiO_3_ and BiFeO_3_ films has been observed to provide continuity of polarization at the interface and circumvent depolarization field issues typical in out-of-plane ferroelectrics, particularly at reduced dimensions.^18^ The underlying Aurivillius phase facilitates the growth of subsequent thin films, ensuring out-of-plane polarization from the very first unit cell. Consequently, it offers depolarizing–field-screening properties and aids in stabilizing ultrathin out-of-plane ferroelectricity in oxide heterostructures.

**Figure 1:**
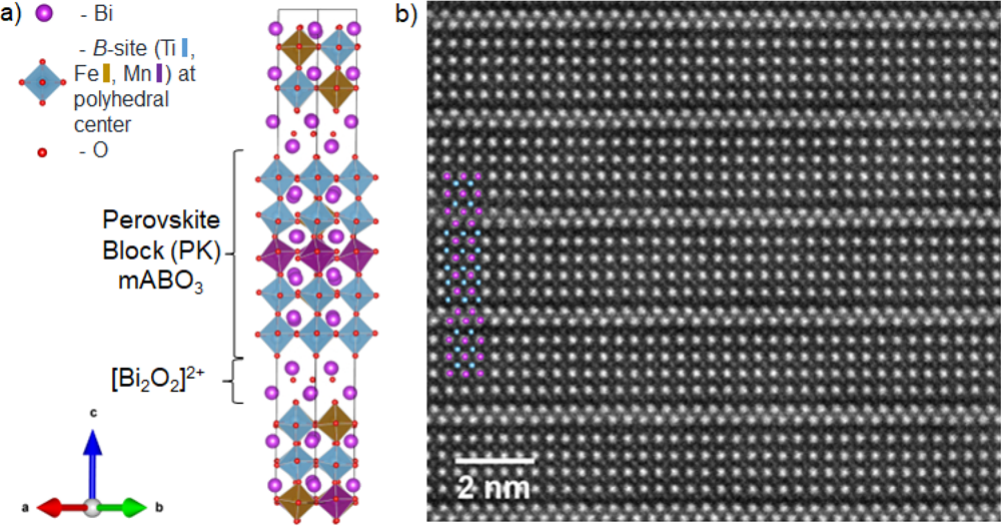
(a) Atomistic structure of one unit cell of the five layered, *m* = 5, B6TFMO ferroelectric structure with B-site displacement and octahedral tilting (as calculated from DFT and discussed as *Configuration: b* in Sections 2.2 and 2.3). Dielectric [Bi_2_O_2_]^2+^ layers are sandwiched between perovskite ABO_3_ units where Bi^3+^ occupies the A-sites (purple) and transition metal cations occupy the B-sites (blue, brown, and purple) of the BO_6_ octahedra. (b) HAADF-STEM image of demonstrating the layering within B6TFMO projected down [110].

When magnetic Fe and Mn cations are integrated into this ferroelectric framework, a unique, stable, single-phase RT multiferroic is formed. As shown in Figure 1, the *m* = 5 phase Bi_6_Ti_*x*_Fe_*y*_Mn_*z*_O_18_ (B6TFMO; *x* = 2.80 to 3.04; *y* = 1.32 to 1.52; *z* = 0.54 to 0.64) has five perovskite layers interleaved between (Bi_2_O_2_)^2+^ fluorite-type layers, while intermixing differing types of A-site (Bi) and B-site (Ti, Mn, Fe) cations within the scaffold drives both ferroelectricity and ferromagnetism/ferrimagnetism (see Supporting Information (SI) Section 1) within the same structural phase.^13−15^ B6TFMO displays saturation magnetization (*M*_S_) values of 215 emu/cm^3^ (two orders of magnitude larger than BiFeO_3_)^7^ and in-plane saturation polarization (Ps) values of >26 μC/cm^2^.^19^ Moreover, B6TFMO demonstrates the reversible linear magnetoelectric switching necessary for practical RT magnetoelectric device applications compatible with low-energy nanoelectronics and spintronics.^13,14^

Inclusion of Ti, Mn, and Fe cations (necessary to achieve the *m* = 5 multiferroic phase) results in a complex bonding environment for the transition metal cations at the perovskite-type B*-*sites of the B6TFMO Aurivillius phase structure (Figure 1). Previous electron microscopy^15^ and density functional theory (DFT)^20^ descriptions of the atomic structure, along with demonstration of the preferred magnetic cation locations (see SI Section 2), has progressed our understanding of the fundamental mechanisms governing long-range magnetic order and net magnetization in this interesting single phase multiferroic. However, it is recognized that functional electric and magnetic properties that drive application in information storage are strongly correlated with octahedral tilting and local structural distortions within perovskite systems and their heterostructures.^12,21−24^ Therefore, tailoring of the B6TFMO composition to enhance multiferroic behavior needs a detailed understanding of atomic-level influences of local symmetry lowering distortions and octahedral tilting on the fundamental electronic structure, ferroelectric properties, and magnetic coupling interactions that arise from differing chemical bonding environments. This knowledge is currently lacking for B6TFMO multiferroics.

Accordingly, this paper aims to fill that crucial gap in understanding the multiferroic behavior of B6TFMO. We describe the use of high-resolution scanning transmission electron microscopy (STEM) in conjunction with electron energy loss spectroscopy (EELS) to spatially probe atomic-resolution electronic structure at the crystallographic perovskite B*-*site sites within the B6TFMO structure. In conjunction with first-principles density functional theory (DFT) calculations of atom resolved partial density of states (PDOS), we correlate trends in crystal field splitting with changes in the extent of polar tetragonal distortion. Insights from integrated differential phase contrast (iDPC) imaging underscore the indispensable role of octahedral tilting as a facilitator for long-range magnetic order within the multiferroic B6TFMO.

## 2.0 Results and Discussion

### 2.1 Electronic Structure Description of Perovskite Layers within B6TFMO

Figure 1a depicts a model of the five-layered multiferroic B6TFMO where a dielectric [Bi_2_O_2_]^2+^ layer and five ABO_3_ perovskite layers correspond to half of a unit cell. HAADF-STEM imaging allows visualization of the atomic structure of within a B6TFMO thin film sample, shown in Figure 1b. In line with other layered materials characterized by high structural anisotropy, Aurivillius phases commonly exhibit out-of-phase boundary defects, intergrowths, and stacking faults.^25^ These structural irregularities pose added complexity. Hence, this study deliberately concentrated on regions within the sample devoid of such defects.

**Figure 2:**
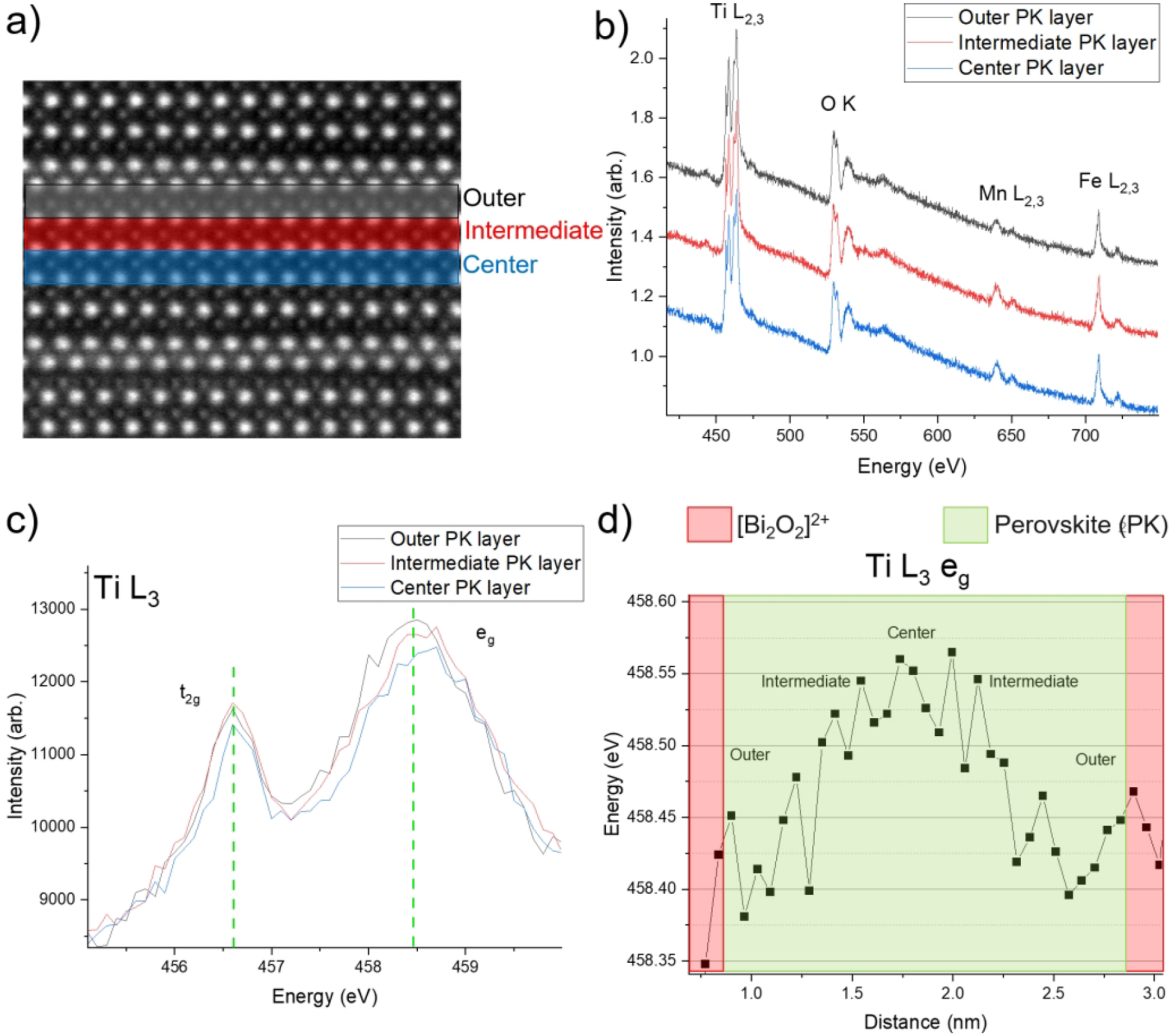
(a) HAADF STEM image of B6TFMO representing the regions outer (black), intermediate (red), and center (blue) from which STEM-EELS spectra were acquired. (b) Normalized EELS spectra representing the three types of perovskite (PK) layers within a B6TFMO unit cell. Annular dark field images were used to extract the EELS spectra. (c) Detail of the Ti L_3_ peak from EELS spectra taken from the three types of perovskite layers. (d) Plot of the change in peak energy of the Ti L_3_ from rebinned EELS spectra. The data set from this figure was rebinned from a 100 × 70 pixel to 1 × 70 pixel data set.

We use spatially resolved EELS (Figure 2) to investigate the local electronic structure of the perovskite sites within its layers. Figure 2b shows the STEM-EELS spectra of the Ti L_2,3_, O K, Mn L_2,3_, and Fe L_2,3_ edges for three different layered regions within B6TFMO’s unit cell: an outer, intermediate, and center perovskite layer (Figure 2a). The outer layers are defined as those closest to the [Bi_2_O_2_]^2+^ interfaces, and the center layers are defined as those furthest from the [Bi_2_O_2_]^2+^ interfaces, with the intermediate referring to the perovskite layers in between. The STEM-EELS measurements of the preferred locations for Ti, Mn, and Fe are in agreement with previous HAADF-STEM EDX (energy-dispersive X-ray analysis) chemical characterization of B6TFMO^15^ (see SI Section 3). While the Mn, Ti, and Fe cations are not fully ordered over the available B-sites and can be found in all five of the perovskite layers, there is a significant preference for magnetic Mn to position toward the center perovskite layers, while there is a distinct preference for Ti to reside at the outer perovskite layers. As demonstrated in Figure S1, the splitting between the t_2g_ and e_g_ peaks of the B-site transition metals is best defined within the Ti L_2,3_ edge. Accordingly, the Ti L_2,3_ edge was selected for further investigation of the electronic structure and subtle chemical bonding changes through the B6TFMO structure, that is, from the [Bi_2_O_2_]^2+^–perovskite interface to the center of the perovskite block.

If the Ti cation is in a well-defined coordination environment, the degeneracy of the 3d orbital final states is lifted due to the crystal field effect arising from hybridization with the occupied 2p orbitals of oxygen. This causes a splitting of the energy levels of the d orbitals. For octahedral complexes, the energies of the t_2g_ interaxial orbitals (d_*xz*_, d_*yz*_, d_*xy*_) decrease (become more stable) and the energy levels of the e_g_ axial orbitals (d_*x2-dx2*_, d_*z2*_) increase owing to the increased repulsion between electrons in the axial 3d orbitals and oxygen 2p. The Ti L_2,3_ edge arises largely from dipole-allowed transitions from the 2p_1/2_ and 2p_3/2_ occupied states into unoccupied 3d energy levels. Crystal field splitting of the 3d orbitals in octahedrally coordinated Ti^4+^ results in a t_2g_-e_g_ splitting of the unoccupied states and thus four peaks are observed in the L_2,3_ edge. For Ti^4+^ cations, the characteristic doublets are well resolved, with clear separation between the t_2g_ and e_g_ peaks. In contrast, if Ti^3+^ was present, the ionization threshold energy would shift to a lower energy, and the four peaks are not clearly observed as a result of the multiplet effects.^26^ Our spatially resolved EELS data (Figure 2c) reveals that the t_2g_-e_g_ crystal field splitting (Δ) is present through the outer, intermediate, and center perovskite layers, indicating an oxidation state of +4 for the Ti cation throughout the B6TFMO structure.

Importantly, layer by layer analysis of the EELS spectra (Figure 2c) taken from the perovskite layers demonstrates an increase in the value of Δ through the layers from the outer layer to center layer. The average increase from the outer perovskite layer to the center perovskite layer is 0.13 ± 0.06 eV. Since this was collected within a single data set, it implies that there is a change in the magnitude of the crystal-field splitting across the five-layered structure. This change in Δ through the layers was observed in two different sample pieces from different DLI-CVD growth runs and measured on different days with two different types of EELS detectors. The spatially resolved EELS spectra demonstrates that the position of the t_2g_ peak position remains constant. Therefore, this change in Δ is due to a shift in the energy of the e_g_ peak, which increases in energy moving from the outer to center perovskite layers. The fact that the t_2g_ level does not change while the e_g_ peak energy position shifts position signifies that this change in the energy levels is associated with a change in the crystal field splitting. Eleven EELS data sets were used to calculate the average Δ value, as shown in SI Table S1. Each data set had two e_g_ values for the outer perovskite layer and one e_g_ value for the center layer; therefore, for each data set, two Δ values were obtained (22 data sets were obtained in total, as shown in SI Table S1). The trend of increased Δ values toward the center layers was observed for all measurements.

Figure 2d and SI Figure S2 display representative plots of e_g_ peak energy as a function of distance through the perovskite layers of B6TFMO, where a definite ascending trend and an energy shift of ∼0.1 eV are observed from the outer to center perovskite layer. The question arises regarding what is responsible for the observed trend in crystal field changes. It is conceivable that several factors that are not mutually exclusive could contribute to crystal field effects and the changes observed in the density of states of the e_g_ energy level. Recent work^21,27^ on (PbTiO_3_)_*n*_/(SrTiO_3_)_*n*_ systems displaying polar vortices has shown changes to the Ti 3d orbital interactions and changes to the crystal field as a function of position within the heterostructure. Given that B6TFMO could be regarded as a natural heterostructure of [Bi_2_O_2_]^2+^ interlayers and Ti[Fe,Mn]O perovskites, in the following sections, we consider possible contributions to the changes that we observe in the Ti L_3_ e_g_ peaks as a function of position. This includes examination of tetragonal distortion differences and variations in tilting of the BO_6_ octahedra imposed by the [Bi_2_O_2_]^2+^ interlayer.

### 2.2 Correlations of Trends in Crystal Field Splitting with BO_6_ Octahedral Distortions and Electrical Polarization at Differing Perovskite Layers within B6TFMO

As determined previously,^15,20,28,29^ the [Bi_2_O_2_]^2+^–perovskite interface within the B6TFMO unit cell drives a change in the internal elastic strain energies and electrostatic energies across the perovskite layers within B6TFMO. Kikuchi’s model^28^ of the Aurivillius phases considers the compressive forces felt at the outer perovskite layers (pseudocubic *a* = 3.89 Å) imposed by the [Bi_2_O_2_]^2+^ interface (*a* = 3.80 Å) and shows how the change in elastic strain energy within Aurivillius phases is critical to the stability of a particular phase. The approach developed by Moore et al.^20^ found that the elastic strain imposed onto the perovskite layers results in the average *c*/*a* ratio changing from ∼1.25 for the outer layers to ∼1.15 at the center layers of the perovskite cells. When considering the impact of elastic strain to the environment within which the Ti cations reside, it follows that the bond lengths between the transition metal and the O ligands will be altered. This ensues that the more highly strained BO_6_ octahedra next to the [Bi_2_O_2_]^2+^ interface will be more tetragonally distorted compared to those further from the interface.

**Figure 3:**
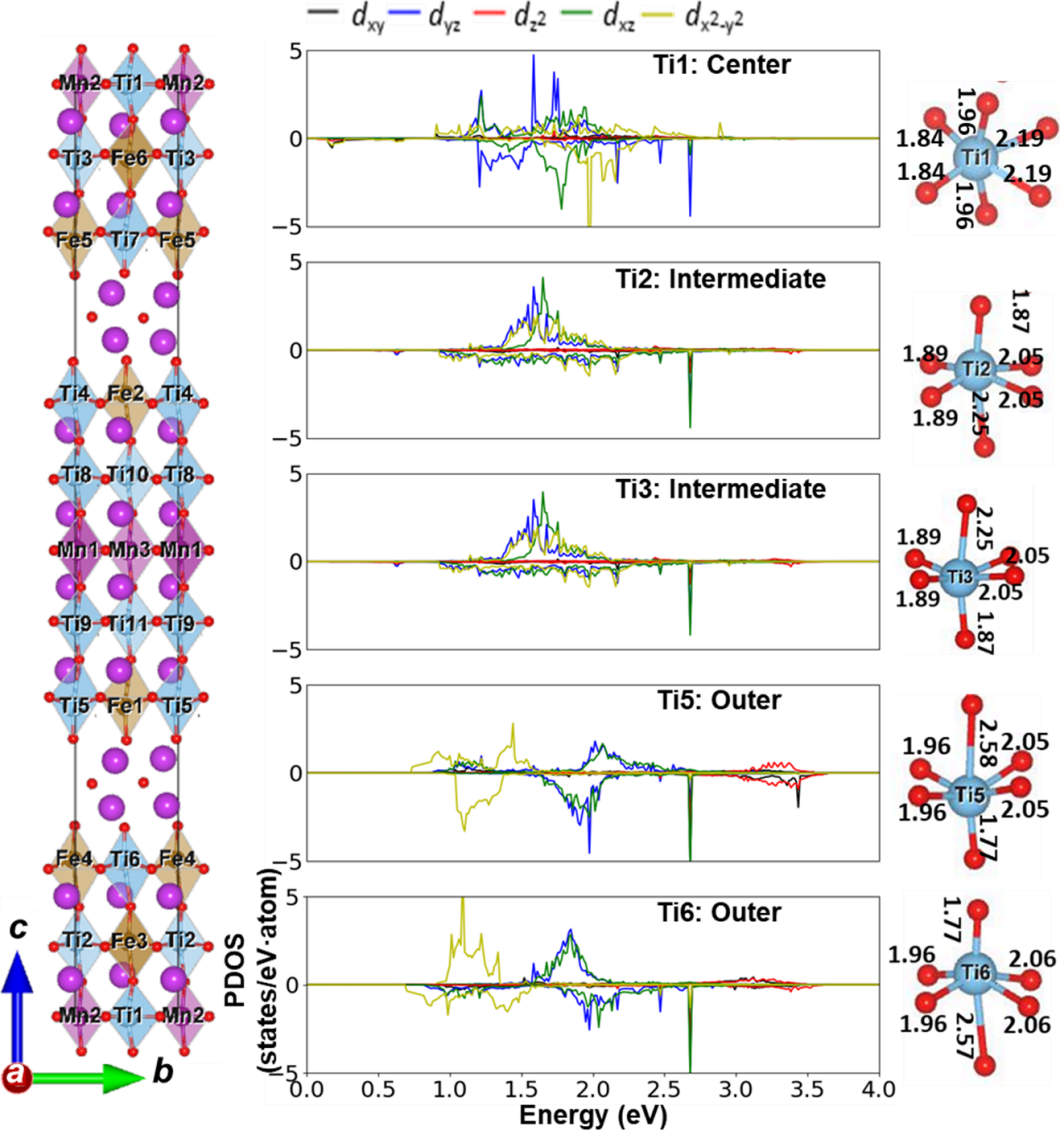
(Left) The fully optimized structure (both lattice parameters and atomic positions) of Bi_24_Ti_11_Fe_6_Mn_3_O_72_ (*Configuration: a*). (Center) The atom-resolved PDOS are shown for selected Ti atoms, where positive (negative) PDOS represents an up (down) spin channel. The PDOS for all the Ti atoms and all the Ti–O bond lengths along the *c*-direction are shown in Figure S4 and Figure S8, respectively, of the Supporting Information. The Fermi energy (EF) is set at 0 eV. (Right) The Ti–O bond lengths are shown in Å units.

To explore the impact of internal elastic strain and tetragonal distortion on local electronic structures, we use first-principles density functional theory (DFT) simulations to analyze the atom-resolved partial density of states (PDOS) in the conduction band region of Ti within a model structure of B6TFMO, which we denote *Configuration: a* (depicted in Figure 3). Although we use DFT to investigate unoccupied state PDOS, earlier studies on Ti-containing perovskites^27^ have shown that the description of the nature of these states to understand EELS spectra with DFT is reliable. Due to its multication composition, numerous potential atomic and magnetic configurations exist for B6TFMO.^20^ For the analysis in this work, we selected *Configuration: a*, based on it having the lowest relative energy among eight different arrangements of Ti, Fe, and Mn atoms over the available B-sites in B6TFMO determined in a prior DFT analysis.^20^ This structure has a clear separation of Fe and Mn sites, while less stable structures show clustering of Fe and Mn, which requires significant local distortions in the atomic structure. *Configuration: a* exhibits Ti cations at the outer, intermediate, and center perovskite layers, facilitating a comparative analysis with STEM-EELS spectra of the Ti L_3_ peaks obtained from these three perovskite layer types (Figure 2).

Our calculated lattice parameters for *a*, *b*, and *c* are 5.48, 5.61, and 51.03 Å, respectively, which are in good agreement with the trend of *b* > *a* from previous experimental XRD measurements of nonepitaxial B6TFMO (*a*, *b*, and *c* of 5.38, 5.43, and 50.26 Å, respectively).^13,14,20^ The slight deviation between DFT calculated and experimentally determined lattice parameters is based on DFT being a zero K, approximate method to evaluate energies and structures within a perfect structure. Furthermore, experimental values are taken from nonepitaxial thin film samples with randomly orientated grains, with stacking fault and out-of-phase boundary defects present. The configuration is in a ferrimagnetic state (see Experimental/Methods) with a net magnetic moment of 15.000 μ_B_ per unit cell (3.75 μ_B_ per formula unit), where Fe1, Fe2, Fe3, Fe4, Fe5, Fe6, Mn1, Mn2, and Mn3 atoms contribute −4.317, −4.317, 4.355, 4.318, 4.318, 4.355, 3.858, −2.919, and 3.846 μ_B_, respectively. The magnetic contribution from the other atoms is minor; for example, the contribution from the Ti1–11 atoms is 0.045, 0.103, 0.103, −0.071, −0.071, 0.077, 0.077, 0.023, 0.023, 0.019, and 0.019 μ_B_, respectively. While DFT analysis of the Bi_5_Ti_3_FeO_15_ Aurivillius phases, in which iron serves as the sole magnetic cation, revealed superexchange-driven antiferromagnetic ordering and a net magnetization of zero,^30^ this present study reinforces earlier DFT studies^20^ of manganese-containing B6TFMO, emphasizing the key role of manganese and the formation of central Mn–O–Mn interactions in promoting ferromagnetic superexchange interactions, long-range ferrimagnetic order, and nonzero net magnetization. Density functional theory + Hubbard (DFT+U) calculations reveal a dominance of the d_*x2-y2*_ orbital around the conduction band minimum of the outer Ti atoms, closest to the [Bi_2_O_2_]^2+^ interface. The d_*x2-y2*_ orbital gradually shifts toward the Fermi level as one progresses from the center (Ti1) to intermediate (Ti2) and outer (Ti4) Ti atoms. Conversely, the d_*z2*_ orbital exhibits an opposite trend, moving toward higher energy. Between the energy range of 3.0 to 3.5 eV, the d_*z2*_ contribution increases from center (Ti1) to intermediate (Ti2) and outer (Ti4) Ti atoms. Our simulations reveal that this occurs due to significant displacements of the outer Ti cations (and also the Fe cations) toward the [Bi_2_O_2_ ]^2+^ interlayers, leading to notable distortions in the TiO_6_ octahedra and the axial Ti–O distances. These types of displacements of transition metal cations toward the [Bi_2_O_2_]^2+^ interlayers are not uncommon for layered perovskite materials and have been observed in other material systems such as the Dion–Jacobson CsANb_2_O_7_ (A = La, Nd, and Bi) phases^31,32^ and Aurivillius phase SrBi_2_Ta_2_O_9_.^33^

Specifically, Ti/Fe/Mn B-site cations located either above or below the center B-site cations of each perovskite block displace in the opposite direction along the *c*-axis, resulting in one short and one long Ti–O distance of 1.77 and 2.56 Å (refer to Figure 3) in the *c*-direction, respectively. This is due to each block being sandwiched between two [Bi_2_O_2_]^2+^ interlayers along the *c*-axis. Consequently, for each five-layered perovskite block, a net zero electric dipole moment (antipolar displacements) along the *c*-axis direction is established if the horizontal mirror plane persists. This antipolar displacement along the *c*-axis direction of B6TFMO reduces the hybridization interaction between the axial d_*x2-y2*_ orbitals of Ti and the 2p orbitals of the oxygen atoms on the *ab* plane. However, it increases the interaction between the axial d_*z2*_ orbitals of Ti and one of the oxygen atoms along the *c*-axis direction. The principal net electric polarization component is created along the *b*-axis (in-plane) direction, originating from each Ti/Fe/Mn atom shifting from its center position, leading to alternative short and long metal–oxygen bonds (see Figure 3) along the *b*-axis. The in-plane ferroelectric displacements are stabilized by the second order Jahn–Teller distortions.

Additionally, a similar orbital shifting pattern is observed for the d_*x2-y2*_ and d_*z2*_ orbitals of the Ti atoms when transitioning from the outer to the intermediate perovskite layers in another B6TFMO model, referred to as *Configuration*: *b* (illustrated in Figure 1a and SI Figure S5). The use of *Configuration*: *b* is justified because the Fe/Mn/Ti distribution mirrors the observations from spatially resolved EDX^15^ and the present EELS results (Figure 2b and SI Figure S1e,f), which demonstrate an increased B-site occupancy (17 to 20%) of magnetic Mn cations toward the center perovskite layers and an increased B-site occupancy of Ti (approximately 20%) cations toward the outer perovskite layers. Our EDX/EELS experiments show that overall, there appears to be a slight preference (3 to 5% increase) for Fe to partition into the center perovskite sites rather than the O sites, but this preference is not significant. These models of *Configuration*: *a* and *Configuration*: *b* facilitate the exploration of different atomic and magnetic configurations of Fe, Mn, and Ti atoms, each displaying varying degrees of octahedral tilting within the same composition, as depicted in SI Figure S5. It should be noted that *Configuration: b* lacks Ti atoms in the center perovskite layer, rendering it unsuitable for a direct comparison with the crystal-field splitting of Ti observed by EELS in Figure 2. Nevertheless, despite these variations, the same antipolar displacement of the intermediate and outer Ti atoms along the *c*-axis direction persists, as shown in SI Figure S8, underscoring the influence of the [Bi_2_O_2_]^2+^ interlayers on these distortions. We propose that this intrinsic orbital position shifting of Ti-derived electronic states across the perovskite block, resulting from the atomic displacement and tetragonal distortion of Ti, constitutes the primary origin of the crystal field splitting shift (Δ) observed within the spatially resolved EELS data of B6TFMO, extending from the outer to the center perovskite layer. It is noteworthy that the relative intensity of the peaks in the L_3_ edge does not change significantly with the change in Δ, which might be expected based on the DOS associated with d_*x2-y2*_ and d_*z2*_ moving apart as indicated in the DFT results. However, the shift to higher energy of the d_*z2*_ orbital is due to increased hybridization with the O_2p_. This will increase the DOS in this energy window and will also increase the transition matrix element, while the opposite effect will occur for the d_*x2-y2*_ orbitals, meaning a marginal net effect on the peak intensities.

The impact of strain and tetragonal distortion imposed by the [Bi_2_O_2_]^2+^ interlayer on the ferroelectric properties can be further observed through local polarization mapping of atomic-scale displacements within B6TFMO by quantifying high-resolution HAADF-STEM signals. Utilizing the full HAADF-STEM image in SI Figure S3a, we calculate the average in-plane displacements of the B*-*site cations along the principal *b*-axis (SI Figure S3b) to be 0.199 ± 0.006, 0.268 ± 0.009, and 0.243 ± 0.009 Å for the outer, intermediate, and center perovskite unit cells, respectively. Comparable magnitudes were noted in B-site displacement values across a distinct region of the sample during analysis conducted on a subsequent day. The in-plane displacement values presented here are comparable in magnitude to those calculated by Campanini et al.^34^ using quantitative analysis of HAADF-STEM data for the *m* = 4 phase, Bi_5_Ti_3_Fe_2_O_15_, wherein *B*-site cation displacement values along the in-plane *b*-axis were observed to be up to ∼0.25 Å. Our DFT+U optimized structures in SI Figure S4 demonstrate corresponding displacements along the *b*-direction. The difference in bond lengths between the alternative short and long metal–oxygen bonds along the in-plane direction reflects the atomic displacement along the *b*-direction, demonstrating similar magnitudes.

As demonstrated in the DFT calculations (Figure 3 and SI Figure S4), the B-site cations within the center perovskite layer of the B6TFMO system are positioned near the center of the pseudocubic perovskite unit cell along the *c*-axis direction, in contrast to the B-site cations in the outer layers, which are displaced toward the [Bi_2_O_2_]^2+^ interface in an antipolar arrangement. The local out-of-plane polarization component within the perovskite unit cells can be measured by quantifying the displacement of the B-site cations in the *c*-axis direction, the strength of which varies as a function of layer. Atomic scale mapping of polar displacements reveals that polarization is most significant toward the outer perovskite cells, correlating with the increased local tetragonal distortion of the octahedral TiO_6_ geometries. From the analysis of SI Figure S3a, it was determined that the average B*-*site cation displacements away from the center of the unit cell along the *c*-axis direction (SI Figure S3b) are 0.426 ± 0.142, 0.184 ± 0.095, and 0.054 ± 0.062 Å for the outer, intermediate, and center perovskite unit cells, respectively. Similar levels of displacement were observed in the B-site values across a distinct region of the sample during analysis carried out on a subsequent day. In addition, similar displacements along the *c*-direction, exhibiting the same overall trend, are observed from our DFT+U optimized structures (see SI Figure S8). Values for the out-of-plane displacement for the outer perovskite layer (0.426 Å) in *m* = 5 B6TFMO are comparable to those found for the perovskite layers next to the [Bi_2_O_2_]^2+^ interfaces in other Aurivillius systems. Newnham et al.^35^ reported out-of-plane B-site cation displacements in the layers next to [Bi_2_O_2_]^2+^of 0.42 and 0.35 Å for the Bi_3_TiNbO_9_ (*m* = 2) and Bi_4_Ti_3_0_12_ (*m* = 3) systems, respectively. Due to the breaking of the horizontal mirror plane within the odd-layered Aurivillius systems (with retention of a 2-fold rotation axis parallel to *a*), a minor net out-of-plane polarization component along the *c*-axis is allowed, in conjunction with the major ferroelectric polarization along the *b-*axis direction.^27,30^ This results in domains within *m* = 5 B6TFMO displaying either a net-up or net-down out-of-plane polarization.^13,36^

### 2.3 Analysis of BO_6_ Octahedral Tilting at Differing Perovskite Layers within B6TFMO

Although it is well-established that functional electric and magnetic properties are profoundly associated with octahedral tilting within perovskite systems,^12,22−24,27^ the space group, the nature of the BO_6_ octahedral tilt system, and the full behavior of the oxygen anions in the relatively new B6TFMO multiferroic system are hitherto unknown. Calculation of the Goldschmidt tolerance factor, *t*,^37^ (see SI Section 4) for B6TFMO yields a value of 0.90. The tolerance factor of <1 indicates that distortion within B6TFMO would be expected^16,29,38,39^ owing to divergences between the A–O and B–O interatomic distances. The ideal high symmetry paraelectric parent phase is the *I*4/*mmm* space group. It has previously been proposed^16,40^ that symmetry lowering in the Aurivillius phases (see SI Section 4) is caused by tilting of the oxygen octahedra around the *ab* plane (tilt mode designated by the irreducible representation (*irrep*) notation *X*_3_^+^), rotations of the oxygen octahedra around the *c*-axis (*irreps X*_2_^+^, *X*_1_^–^), (shown in SI Figure S6), and the polar cation motions along the *b*-axis (*irrep* Γ_5_^–^), which when coupled together contribute to a material’s ferroelectric ground state. The B6TFMO structure, which is orthorhombic, √2 a_t_ × √2 a_t_ × c_t_ (where t denotes the high symmetry parent structure of *I*4/*mmm* symmetry), has not yet got an experimentally measured space group and could potentially be described by ferroelectric space groups such as *F2 mm (irrep* Γ_5_^–^)^41^*or B*2*eb* (*irreps* Γ_5_^–^, *X*_3_^+^).^38^ The *B*2*eb* space group does not possess inversion symmetry, and structures belonging to this space group are potential candidates for exhibiting direct linear magnetoelectric coupling. Neutron diffraction has been typically used to obtain information about the nature of the structural ordering in the Aurivillius phases. However, diffraction techniques lack the spatial resolution required to establish links between the atomic scale structure, chemical composition, and bonding environments concerning the origins of multiferroic properties.

Integrated differential phase contrast STEM (iDPC-STEM) in conjunction with HAADF-STEM can be used for the direct atomic resolution visualization of light atoms at sub-Å resolution.^42^ The technique allows for the observation of low *Z*-elements, such as oxygen, using a bright contrast and dark background with a higher signal-to-noise ratio when compared to competing techniques such as annular bright field STEM (ABF-STEM).^43^ As STEM imaging is inherently two-dimensional, determining a definitive space group for B6TFMO using this characterization technique is challenging. However, information about the BO_6_ tilting can be gained by imaging the system in multiple crystal orientations. In this study, iDPC-STEM imaging is employed to visualize the distribution of light oxygen atoms across different layers within the Aurivillius phase. This approach provides a deeper insight into their bonding environments within the perovskite layers.

**Figure 4:**
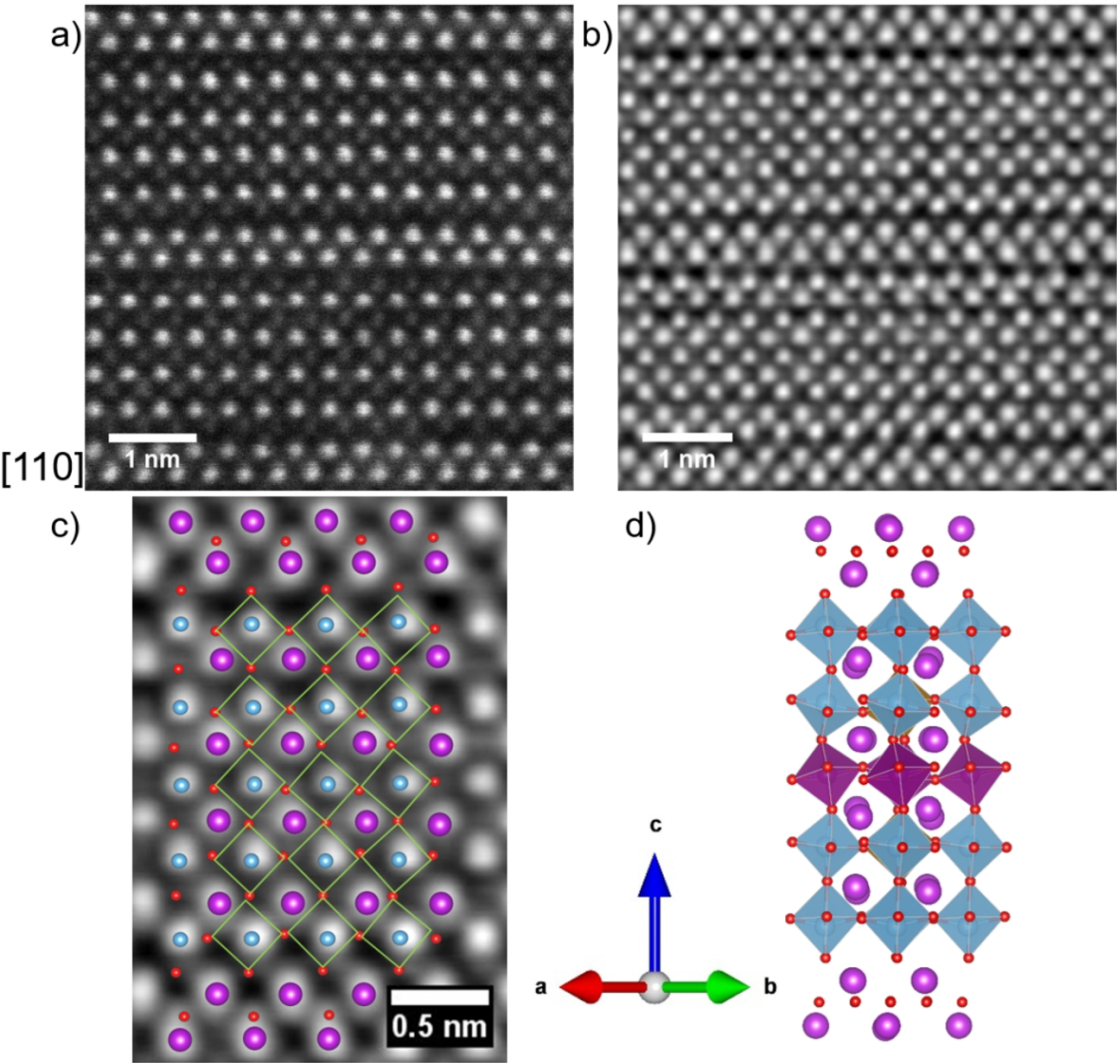
(a) HAADF-STEM and (b) iDPC-STEM images of B6TFMO projected down [110]. (c) Enlarged region where identifiers for atoms and polyhedra are overlaid, (d) comparison of the five-layered Aurivillius phase calculated from DFT+U in *Configuration*: *b* that corresponds to the atomic arrangement (i) shown in SI Figure S7c, but when projected down [110]. Bi^3+^ cations are represented by purple spheres, oxygen anions are represented by red spheres, and B-site transition metal cations are represented by blue spheres or blue (Ti) /purple (Mn) /brown (Fe) octahedra. Octahedral tilting is mostly hidden when viewed down the [110] projection.

**Figure 5:**
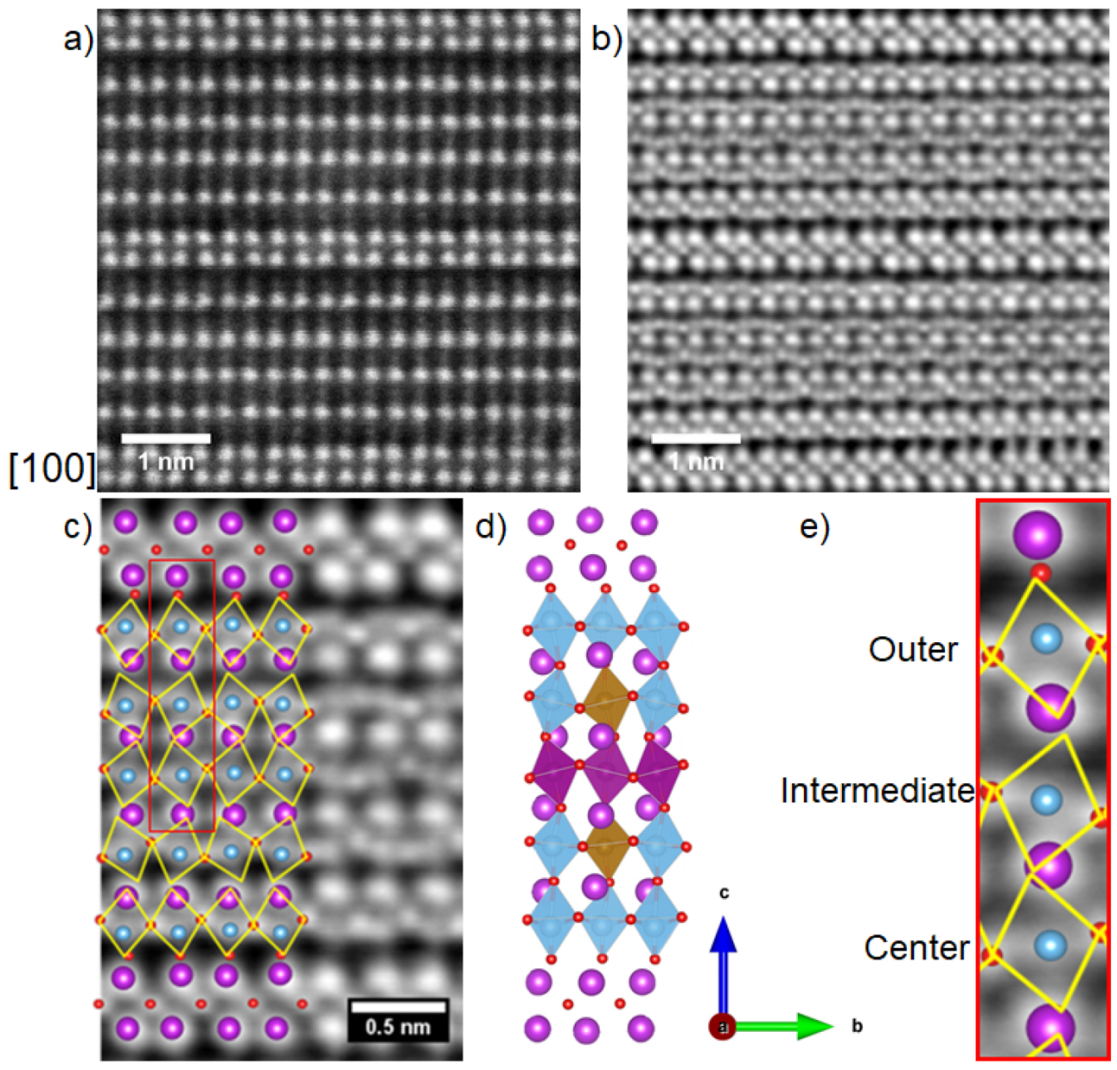
(a) HAADF-STEM and (b) iDPC-STEM images of B6TFMO projected down [100]. (c) Enlarged region where identifiers for atoms and polyhedra are overlaid onto the image. (d) Comparison to the DFT structure corresponding to arrangement (i) in SI Figure S7c projected down [100]. This image illustrates why octahedral tilting is more clearly observed in the iDPC-STEM images when projected down [100] compared with down [110]. (e) Enlarged region within the red inset from (c) displaying increased tilting of the octahedra moving from the outer to center perovskite layers. Bi^3+^ cations are represented by purple spheres, oxygen anions are represented by red spheres, and B-site transition metal cations are represented by blue spheres or blue (Ti) /purple (Mn) /brown (Fe) octahedra.

iDPC-STEM measurements of the positions of the oxygen anions and their relationship to the Bi and B-site cations are shown in Figure 4 and Figure 5, viewed down the [110] projection (Figure 4) and down the [100] projection (Figure 5). One can observe the impact of the [Bi_2_O_2_]^2+^ interface on B–O distances and corresponding displacements of the B-site cations. The close-up in Figure 5e enables us to visualize the formation of a short bond between the bismuth cation in the [Bi_2_O_2_]^2+^ interface layer and an apex oxygen of the adjacent (outer) perovskite layer.^35^ This influences the B–O bond distances in the *c*-axis direction and prompts a polar displacement of the B-site cations and ferroelectricity along the *b*-direction. The displacement lessens as we move toward the center perovskite layer, aligning with the DFT results presented in Figure 3 and SI Figures S4 and S8 and is consistent with the systematic change in the crystal field splitting observed in the EELS measurements.

Comparisons between DFT models of the identical B6TFMO configuration, as depicted in the [110] projection (Figure 4d) and the [100] projection (Figure 5d), reveal that the tilted nature of the oxygen atoms in the equatorial plane is largely obscured when observing from the [110] perspective. This concealment is attributed to the alternating pattern of octahedral tilting and the overlapping arrangement of oxygen atoms parallel to the *b-*axis. This stands in contrast to observations from the [100] projection, where cooperative tilting of BO_6_ octahedra within the B6TFMO structure is distinctly evident in both DFT models (Figure 5d) and STEM-iDPC images (Figure 5b,c,e). Through an examination of oxygen positions and a comparative analysis with existing literature,^16,40^ it becomes apparent that a tilt mode akin to the irreproducible representation (*irrep*) *X*_3_^+^ (SI Figure S6) is present. This mode accounts for the in-plane (perpendicular to *c*) antiphase tilting of octahedra with respect to each other in the *ab* plane.

The average degree of the octahedral tilting is approximated based on data extracted from Figure 5c,e and SI Figure S7, with corresponding values displayed in SI Table S2. The findings from this STEM-iDPC investigation of B6TFMO, along with neutron diffraction data collected from other *m* = 5 Aurivillius phase systems in the literature^38^ (as illustrated in SI Figure S7), collectively indicate an escalating degree of tilting progressing toward the center perovskite layer. The observed increase in tilting through the layers toward the center in B6TFMO hints at a rotational movement of the inner octahedral layers only around the *c*-axis, indicating the possible presence of the *irrep X*_2_^–^ mode at the center, in addition to the *irrep X*_3_^+^ tilt mode (SI Figure S6). However, further analysis of the structure through neutron diffraction or HAADF-STEM imaging along different axes is required to corroborate this. The B6TFMO system manifests a relatively substantial tilting of the oxygen atoms, with approximate tilt angles for the octahedra along ⟨010⟩ being ∼5° for outer layers, ∼11° for intermediate layers, and ∼16° for center layers. This complex octahedral tilting, characterized by variable tilt amplitudes across the perovskite layers, competes with the extent of tetragonal distortion observed in the TiO_6_ octahedra. Notably, the outer TiO_6_ octahedra, positioned adjacent to the [Bi_2_O_2_]^2+^ layers exhibit the least rotation, maintaining a more tetragonally distorted geometry. Significantly, tetragonal distortion is minimized, and the degree of octahedral tilting is maximized at the center layers of B6TFMO, which serve as the preferred partitioning sites for magnetic cations. We employ the identical methodology employed for the experimental data presented in Figure 5c and SI Figure S7a to approximate the tilt angle values along the in-plane axis for the structures simulated via DFT+U calculations. Two distinct trends are observed between *Configuration*: *a* and *Configuration*: *b* (refer to SI Table S2). Unlike *Configuration*: *b*, *Configuration*: *a* does not exhibit the same increasing trend of tilt angles toward the center perovskite layers as evident in our experimental iDPC data and the neutron diffraction data reported by Ismunandar et al.^38^ Our analysis reveals comparatively minor in-plane tilting along ⟨010⟩ for *Configuration*: *a*. Additionally, we observe that octahedra in *Configuration*: *a* do not exhibit the alternating tilting pattern along the *b*-axis characteristic of the *irrep X*_3_^+^tilt mode (SI Figure S6). This deviation contrasts with the experimental data obtained from the structures examined (refer to SI Table S2).

*Configuration*: *b,* however, exhibits evidence of tilting resembling the *irrep X*_3_^+^ tilt mode and a trend akin to the experimental data. We observe that the degree of tilting increases as we traverse from the outer Ti atoms to the center Ti atoms. For instance, consider the five perovskite layers depicted in column 1 of arrangement (i) in SI Figure 7 (c) (corresponding to the center of *Configuration*: *b* in SI Figure S8 and SI Figure S9). Progressing down the *c*-axis from the top [Bi_2_O_2_]^2+^ layer to the layers below, the tilt angle values along the in-plane axis vary as follows: −6° for outer (top), + 13° for intermediate (top), −14° for center, + 6° for intermediate (bottom) and +2° for outer (bottom). Due to the dynamic arrangement of Ti, Fe and Mn cations in *Configuration*: *b*, the measured tilt values fluctuate depending on cation arrangement. The range of tilt values for the outer, intermediate, and center octahedra is ∼2 to 6°, ∼ 1 to 13° and ∼6 to 14° respectively. It is evident across all atomic arrangements within *Configuration*: *b* that the magnitude of tilt angle at the outer *BO*_*6*_ octahedra is consistently smaller than that at the center *BO*_*6*_ octahedra, aligning with the observed tilting trends in the experimental data (refer to SI Table 2). In *Configuration*: *b*, some of the outer and intermediate tilt angles are comparable to, if not greater than, those observed for the A_2_Bi_4_Ti_5_O_18_*m* = 5 Aurivillius systems previously characterized^38^ by neutron diffraction, as illustrated in SI Table S2.

The slightly larger tilting observed in our experimental iDPC data and DFT *Configuration*: *b* aligns with the tolerance factor for the B6TFMO composition (*t* = 0.90), which is lower than that of A_2_Bi_4_Ti_5_O_18_.^38^ Furthermore, our investigations indicate that it is necessary for the B6TFMO structure to adapt to accommodate varying arrangements of *B-*site atoms, manifesting in distinct magnitudes of octahedral tilting for different B6TFMO configurations. In *Configuration*: *b* we note instances where FeO_6_ and MnO_6_ octahedra are adjacent in a perovskite layer (e.g., SI Figure S7 (c)), whereas in *Configuration*: *a*, FeO_6_ and MnO_6_ octahedra are spaced further apart (e.g., SI Figure S7 (b)). From *Configuration*: *b,* it becomes evident that when Fe and Mn are positioned next to each other in the structure, a more pronounced octahedral tilting occurs across the perovskite layers. This phenomenon may result from the structure adapting to accommodate varying metal–oxygen distances in Ti–O, Fe–O and Mn–O. For example, along the *c*-axis direction in *Configuration*: *b*, the longest Ti6–O, Fe2–O, and Mn1–O bonds measure 2.50 Å, 2.52 Å, and 1.99 Å, respectively (SI Figure S8 illustrates how these bond lengths vary along the *c*-axis direction in both configurations).

Consistent with prior literature^29,38^ on the Aurivillius phases, our calculations demonstrate that octahedral tilting contributes to the minimization of the electrostatic potential and stabilization of the material when FeO_6_ and MnO_6_ octahedra are located close to each other. A previous DFT study^20^ investigated the energetics of eight configurations of the B6TFMO Aurivillius phase. The highest relative energy was +3.24 eV for a composition where the iron and manganese cations are clustered together which requires significant octahedral tilting and stretching. The lowest-energy configuration (with a relative energy of 0 meV) was observed when manganese positions within the center perovskite units and where the iron and manganese cations are separated, corresponding to *Configuration: a* from the present study (Figure 3). *Configuration*: *b* (SI Figure S5) displayed a slightly higher relative energy of +10 meV. *Configuration*: *b* corresponds with the EDX^15^ and EELS experimental observations of magnetic cation partitioning within B6TFMO, where manganese shows a preference for locating to the center perovskite units and where the iron cations a slight preference toward the center and intermediate perovskite layers. As discussed above, the manganese and iron cations within *Configuration: b* are not as well-separated compared with *Configuration: a*, therefore *Configuration: b* requires an increased degree of octahedral tilting as compared with *Configuration: a*. For *Configuration: b*, the extent of octahedral tilting competes with the extent of tetragonal distortion. This octahedral tilting is observed experimentally in the iDPC-STEM images in Figure 5.

In addition to manifesting significantly distinct magnitudes of octahedral tilt angles, our two configurations, as determined by DFT+U calculations, exhibit varying deviations in bond angle from 180° along the *c*-axis direction. The accurate measurement of oxygen and *B*-site positions in the DFT simulated structures is facilitated, with SI Figure S8 illustrating bond angles at each *B*-site cation between two *B*-O bonds, and SI Figure S9 depicting bond angles at each oxygen (O)-site between two *B*-O bonds. However, despite this, *Configuration*: *a* and *Configuration*: *b* display analogous antipolar atomic displacements of Ti cations, with the extent of tetragonal distortion competing with the extent of octahedral tilting, consistent with experimental data. Furthermore, similar *d*_*x2-y2*_ and *d*_*z2*_ orbital shifting is observed for the two configurations within the atom resolved PDOS data (Figure 3, SI Figure S4 and SI Figure S5). Therefore, we posit that octahedral tilting does not exert a significant influence on crystal field splitting. Our proposal is substantiated by the similarity in the observed effects on crystal field splitting, emphasizing that the primary driving factor for changes in the *L*_3_*e*_g_ EELS peak from the outer to center perovskite layer is the atomic displacement and tetragonal distortion of Ti, influenced by its position within the Aurivillius unit cell. This relationship is expected to remain consistent across all potential atomic configurations of the five-layered B6TFMO structure.

Given that the highest degree of octahedral tilting occurs in the center layers where the magnetic cations preferentially partition, exploring the implications of this tilting on the magnetic properties of B6TFMO becomes intriguing. Our calculations determine a net magnetic moment for *Configuration*: *b* (33.892 μ_B_ per unit cell), characterized by larger tilt amplitudes at the center layer, more than two times higher than that calculated for *Configuration*: *a* (15.000 μ_B_ per unit cell). However, it is essential to note that *Configuration*: *a* and *Configuration*: *b* exhibit distinct distributions of *B*-site cations, and as established from previous studies,^20^ magnetization values are highly dependent on cation site positioning. From these examples, it is not possible to deconvolute the individual contributions of octahedral tilting and cation site positioning to the resultant magnetic moment. While octahedral tilting itself may not be the primary driver of magnetism in B6TFMO, it becomes evident that tilting of the perovskite structure is necessary to facilitate the proximity of magnetic iron and manganese cations within the center perovskite layers. Consequently, perovskite octahedral tilting emerges as a vital requirement for enabling long-range magnetic interactions and, subsequently, the materialization of multiferroic behavior in B6TFMO.

## Conclusions

The B6TFMO material, characterized by its natural layering and room temperature multiferroic properties, as well as distinctive topological features,^13,14,20^ exhibits significant potential for applications in nanoelectronics and spintronics. This paper has delved into understanding the mechanisms underlying multiferroic behavior, crucial for practical applications, by investigating the impact of local symmetry-lowering distortions and octahedral tilting on polar and magnetic functionalities within B6TFMO. Employing atomic-resolution electron microscopy in conjunction with first-principles calculations, we have correlated the fundamental electronic structure with the local chemical bonding environments within B6TFMO.

Spatially resolved HAADF-STEM EELS has detected discrete changes in the crystal field of the Ti^4+^ cations, revealing an increase in e_g_-t_2g_ splitting, Δ, from the outer to the center perovskite layers of the structure. DFT calculations, incorporating Hubbard U corrections of the atom-resolved projected density of states, demonstrates that the distortion of the perovskite unit cells, induced by their proximity to the fluorite-type [Bi_2_O_2_]^2+^ interlayers, is the main factor impacting the e_g_ energy levels and crystal field splitting changes. Atomic-resolution HAADF-STEM imaging corroborates antipolar atomic displacements of the Ti cations influenced by the [Bi_2_O_2_]^2+^ interlayers, demonstrating that polarization is most pronounced toward the outer perovskite cells, coupled with a significant increase in tetragonal distortion of the octahedral TiO_6_ geometries in these outer layers.

HAADF-iDPC imaging and DFT calculations unveil a complex cooperative tilting of the BO_6_ perovskite octahedra, competing with the extent of tetragonal distortion across the perovskite layers. Tetragonal distortion is minimized, and the degree of octahedral tilting is maximal at the center layers of B6TFMO, coinciding with the highest concentration of magnetic cations. Theoretical calculations show that changes in the cooperative octahedral tilting contribute to minimizing the electrostatic potential and stabilizing the material. However, the extent of crystal field splitting remains independent of the distribution of tilt angles and magnetization in the supercell, confirming that changes to Δ are primarily driven by the introduction of tetragonal distortions at the fluorite–perovskite interface.

Observations from both iDPC experiments and DFT simulations reveal that perovskite octahedra need to tilt more at the center layers to accommodate closely positioned magnetic cations, signifying that octahedral tilting might play a crucial role in enabling long-range magnetic interactions and the resultant multiferroic behavior in B6TFMO. Our findings not only enhance the understanding of the naturally layered B6TFMO but also propel multiferroic materials science to the forefront. They highlight that engineering interfaces in heterostructures and layered materials presents a promising avenue for controlling material properties, marking transformative strides in multifunctional material design and application.

## Experimental/Methods

### Thin Film Growth

Direct liquid injection chemical vapor deposition (DLI-CVD) methods similar to Faraz et al.^14^ were used to deposit B6TFMO thin films on *c*-sapphire. Samples from two different growth runs were synthesized by DLI-CVD via a horizontal flow AIXTRON AIX 200/4FE AVD (atomic vapor deposition) system.^44^ The films had a nominal stoichiometry of Bi_6_Ti_3_Fe_1.5_Mn_0.5_O_18_. The films were deposited using a 22 to 23% bismuth excess^45^ to mitigate the loss of volatile bismuth^46^ during the two-step growth process.^47^ The 0.100 mol dm^–3^ bismuth, iron and titanium precursors in toluene solution, Bi(thd)_3_, Fe(thd)_3_, and Ti(O-iPr)_2_(thd)_2_ (where thd = 2,2,6,6-tetramethyl 3,5-heptanedionate and O-iPr = isopropoxide), were purchased from Epivalence Ltd. The 0.025 mol dm^–3^ manganese precursor was prepared by dissolving Mn(thd)_3_ (99%, STREM chemicals) in anhydrous toluene (99.8%, Sigma-Aldrich) under N_2_ in a glovebox. The substrate was loaded into the reactor chamber set at a pressure of 10 mbar, while the temperature of the rotating susceptor-heated sample holder was set to 630 °C. The conditions were maintained for the entirety of the deposition process. The low vapor pressure precursors were then injected using nitrogen pulses into the Trijet Vaporizer of the DLI-CVD system, where they entered a reactor growth chamber via lines maintained at 220 °C. Precursors were mixed with reactive O_2_ gas during the growth run. A total gas flow of 3000 sccm (standard cubic centimeters per minute) was maintained in the growth chamber comprised O_2_ run gas and N_2_ carrier gas where the O_2_ flow was 1000 sccm. The precursor volumes were controlled electronically by setting the opening time for each injection to be between 1.6 and 10.9 ms, depending on the precursor and using the continuous injection mode where the injections pulsed at a frequency of 1 Hz. A total of 800 injections of each precursor were used, and the net volumetric precursor injection ratios were approximately 7.4:3:1.5:0.5 for Bi(thd)_3_, Ti(O-iPr)2(thd)_2_, Mn(thd)_3_, and Fe(thd)_3_, respectively, over the duration of the growth run. After thin film deposition via DLI-CVD (step one), the samples were postannealed in an atmosphere for 1 h at 850 °C (step two) to produce crystalline Aurivillius phase films. The thicknesses of the subsequent B6TFMO films were approximately 95 nm.

### Electron Microscopy

A FEI DualBeam Helios NanoLab 600i Focused Ion Beam instrument was used to prepare the lamella cross sections of the B6TFMO films. Transition electron microscopy (TEM) cross sections were prepared by depositing C and Pt as electron-beam induced layers followed by an ion-beam induced C layer for protection. The lamellas were thinned at 0.28 nA/30 kV with final polish at 47pA/5 kV. High angle annular dark field (HAADF)-scanning transmission electron microscopy (STEM) imaging and integrated differential phase contrast (iDPC)-STEM imaging were performed using a probe-corrected Thermo Fisher Scientific Themis Z operated at 200 kV with a convergence semiangle of 18 mrad. iDPC-STEM images were obtained using a four-segment annular detector with a hole in the center. ADF images were collected simultaneously with an annular dark field detector for correlation. A live high-pass filter was applied to iDPC-STEM images to reduce the low frequency information.

Electron energy loss spectroscopy (EELS) spectrum imaging (SI) was performed at 300 kV using an image-corrected Titan3 G2 60-300 with a monochromated Schottky-FEG and a Gatan Quantum with both an UltraScan 1000 CCD and a K2 direct electron detector. EELS was acquired in STEM mode with collection (β) to convergence (α) angle ratios of β/α = 1–2. No significant variations were observed with a larger β/α ratio tested to improve the S/N. Data was collected with a dispersion of 0.1 eV/channel and an energy resolution between 0.3 and 0.4 eV determined from the full width at half-maximum of the zero-loss peak. Dual-EELS was used for energy shift correction but was not available with the K2.

A rebin function using Gatan GMS software was utilized to try and improve the signal:noise ratio of the EELS data. This function consists of summing the intensities of neighboring pixels into a single pixel. The number of pixels within the data set is subsequently reduced. In this analysis, the horizontal (*x*-component) of a data set was summed into one pixel and the number of vertical (*y*-components) was not changed. For example, in Figure 2, the data set was rebinned from a 100 × 70 pixel to a 1 × 70 pixel data set. The data for the change in peak energy of the Ti L_3_ from rebinned EELS spectra was calculated using Gatan’s Digital Micrograph EELS module. For each data set, nonlinear least-squares (NLLS) fitting (Gaussian and Lorentzian) in Gatan’s Digital Micrograph EELS module was used on the Ti L_2,3_ edge. The energy positions (eV) of Ti L_2,3_ peaks were then plotted as a function of distance through the perovskite layers. The average shift of the Ti L_3_ e_g_ peak was calculated by utilizing the EELS spectra of the outer and center perovskite layers using annular dark field images. The Ti L_2,3_ peak values were obtained using multipeak fit in origin. The atomic scale polarization mapping was carried out on STEM-HAADF images by first using Atomap software^48^ that analyzes the positions of atomic columns using 2D elliptical Gaussian distributions. Once the A- and B-site positions of the atoms are determined, the polarization vectors are found by reversing the measured B-site displacement, using the TEMUL toolkit.^49^

### Density Functional Theory Calculations

We performed spin-polarized density functional theory (DFT) calculations using the Vienna Ab initio Simulation Package (VASP5.4).^50^ The ion–electron interactions were described by the projector augmented wave (PAW) methods.^51^ The exchange and correlation effects were described by the Perdew–Burke–Ernzerhof (PBE) functional of the generalized gradient approximation (GGA).^52,53^ A rotationally invariant Hubbard U correction^54^ was added to the 3d states of Fe and Mn, where *U* = 5.5 eV was used for all Fe atoms (the typical range of U values for Fe in multiferroic BiFeO_3_ is between 5 and 7 eV^55,56^) and 3.0 eV was used for Mn atoms.^20^ The *U* value is not required for Ti, whose PDOS is examined in this study to theoretically rationalize the experimental EELS spectra of Ti. Structure files (*Configuration: a* and *Configuration: b*) were taken from previous work,^20^ and while we use a different Hubbard U value for Fe, no significant structural differences are observed. Atom-resolved PDOS data for Ti atoms of *Configuration*: *a* is presented in Figure S10 of the Supporting Information, with *U* values set at 4.0 eV for Fe and 3.0 eV for Mn.^20^ Overall, our analysis reveals that the structural parameters and electronic properties exhibit consistency, emphasizing the robustness of our conclusions across various *U* values in the investigated system. The number of valence electrons for Bi, Ti, Fe, Mn, and O is 5 (valence electron configuration: 6s^2^ 6p^3^), 4 (valence electron configuration: 3d^3^ 4s^1^), 8 (valence electron configuration: 3d^7^ 4s^1^), 7 (valence electron configuration: 3d^6^ 4s^1^), and 6 (valence electron configuration: 2s^2^p^4^), respectively. The plane-wave expansion was truncated at a cutoff energy of 600 eV. Similar to the previous DFT calculations,^20^ we use a unit cell of Bi_24_Ti_11_Fe_6_Mn_3_O_72_ stoichiometry. To mitigate the heavy computational burden, we arrange Fe and Mn atoms in an ordered manner within the simplest 1 × 1 × 1 unit cell, comprising 116 atoms. Consistent with previous experimental observations of cation partitioning, as well as theoretical studies,^20^ we consider two Mn atoms at the inner perovskite block. The convergence threshold of energy for the self-consistent calculations was 10^–6^ eV. The structural optimization was carried out by minimizing forces on all the atoms until they were smaller than 0.01 eV Å^–1^. Energy comparisons between different magnetic spin configurations were previously presented.^20^ Ferromagnetic, ferrimagnetic, and three distinct antiferromagnetic orderings were calculated for cation ordering configurations, with the final lowest-energy magnetic order relaxing to a ferrimagnetic case for each configuration. We focus on *Configuration: a* and *Configuration: b*, which were identified as the two lowest energy configurations compared to the others discussed in ref (20). The relaxed atomic *Configuration: a* and *Configuration: b* possess distinct space groups of *Pmm*2 (group number: 25) and *Pm* (group number: 6), respectively. The differences arise due to their differing Fe and Mn atomic configurations, leading to differences in symmetry breaking. A 4 × 4 × 2 Monkhorst–Pack *k*-point mesh^57^ was used for structural optimization and a 6 × 6 × 2 Monkhorst–Pack *k*-point mesh was used for the DOS calculations. The use of symmetry is switched off using ISYM = −1 for both structural optimizations and DOS calculations. Turning off symmetry in the calculation allows for full electronic, atomic, and lattice relaxation so that the systems can relax to the lowest structure, without any constraints introduced by an imposed symmetry. The Gaussian Fermi smearing method (smearing width of 0.01 eV) with 2001 energy bins was used for DOS calculations. Atomistic structures are visualized using the VESTA 3D visualization program.^58^

## Supporting Information Available

Details on magnetic order types of B6TFMO, magnetic cation partitioning within the B6TFMO Aurivillius phases and octahedral tilting in Aurivillius phase systems, additional electron microscopy and density functional theory data, further information on analysis of STEM-EELS spectra (PDF)
