# Supplementary material for: Tilting and Distortion in the Multiferroic Aurivillius Phase Bi6Ti3Fe1.5Mn0.5O18
Source: Chem Mater. 2024 May 29;36(11):5474–86. doi: 10.1021/acs.chemmater.4c00413 (PMC11170937; doi:10.1021/acs.chemmater.4c00413)
Supplement: Supplementary file 1 — cm4c00413_si_001.pdf [file cm4c00413_si_001.pdf]

Supporting Information for:

Tilting and distortion in the multiferroic Aurivillius phase $Bi_6Ti_3Fe_{1.5}Mn_{0.5}O_{18}$

*Louise Colfer,[1] Núria Bagués,[2,3] Mohammad Noor-A-Alam,[1] Michael Schmidt,[1] Michael Nolan,[1] David W. McComb,[2,3] Lynette Keeney[1]**

1. Tyndall National Institute, University College Cork, Lee Maltings Complex, Dyke Parade, Cork, T12 R5CP, Ireland

2. Center for Electron Microscopy and Analysis, The Ohio State University, Columbus, OH, USA

3. Department of Materials Sciences and Engineering, The Ohio State University, Columbus, OH, USA

1. Magnetic Order Types of the B6TFMO Aurivillius Phases

The magnetic properties of B6TFMO have been characterized in previous studies[1,2]. Although there is an increased concentration of magnetic cations at the center-most *B*-sites[3], the complex nature of B6TFMO and the overall disorder in cation distribution pose challenges in definitively determining its magnetic order type — whether it is ferromagnetic, ferrimagnetic or a combination of both. Previous discussions[1–4] have explored potential pathways to ferromagnetic order and the role of manganese in promoting ferromagnetic ordering.

Possibilities for ferrimagnetic order may arise from cation disorder (e.g., configurations analyzed by density functional theory in this study and referenced work[4]), potential uncompensated antiferromagnetically coupled spins, or variations in the valence states of magnetic cations. These scenarios would be reflected in unsaturated magnetization-magnetic field (MH) hysteresis loops, distinct from the saturated MH loops observed in ferromagnets.

The primary challenge in experimentally distinguishing between ferromagnetism and ferrimagnetism lies in the relatively low magnetic moment of the B6TFMO thin films compared to the diamagnetic background originating from the substrate. This complicates the accurate estimation of small differences in saturation and unsaturation. Nevertheless, the maximum observed moment from experiments at a field strength of 5 T and a temperature of 5K is 3.01 μB/formula units (f.u.) (19 emu/cc)[1], which is close to the range of theoretical moments calculated for ferromagnetic alignment, spanning from 3.16 to 8.34 μB/f.u. (depending on the valences and high-spin or low-spin configuration of Mn^{3+}/Mn^{4+} and Fe^{3+}). Consequently, in previous experimental analyses, B6TFMO was designated as ferromagnetic based on these findings.

2. Magnetic Cation Partitioning within the B6TFMO Aurivillius Phases

Below the ferroelectric transition temperature, a strong bond is formed between the bismuth cation in the $[Bi_2O_2]^{2+}$ interface layer and an apex oxygen of the adjacent perovskite layer within the Aurivillius structures. As well as having an inductive effect on the *B-O* bond distances in the layer direction (*c*-axis) [5] and prompting ferroelectricity via a polar displacement of ions perpendicular to the layer direction, the shorter *Bi-O* bond (e.g. 2.42 Å compared with 2.65 Å in $Bi_4Ti_3O_{12}$) shears the perovskite layer, resulting in tilting and rotation of the BO_6 octahedra [6,7]. The presence of the $[Bi_2O_2]^{2+}$ interface layers interleaved within the structure imparts an elastic strain energy gradient through the Aurivillius layers, due to layer mismatch between the differing lateral dimensions of the $[Bi_2O_2]^{2+}$ layers (a = 3.80 Å) compared to the perovskite lattice layers (a = 3.89 Å) [8]. In addition, the layer of oxygen anions in the $[Bi_2O_2]^{2+}$ interface layer provides electrostatic energy variations within the Aurivillius structure. For the case of B6TFMO, substitution of Ti/Fe with magnetic Mn and Fe cations (necessary to achieve the m = 5 multiferroic phase), results in an even more complex bonding environment for the transition metal cations at the available perovskite-type *B*-sites of the Aurivillius phase structure (see **Figure 1** of the Main Text). Atomic resolution energy dispersive X-ray analysis studies have revealed that cation partitioning occurs in B6TFMO due to elastic strain and electrostatic energy contributions, which vary as a function of distance from the $[Bi_2O_2]^{2+}$ fluorite-type layers [3,8–12]. This partitioning is revealed as a preference for Mn to locate predominantly in the center perovskite layers within the five-layered perovskite block enabling an increase in the probability of nearest-neighbor magnetic interactions in the center layer by up to 90 % compared to a scenario where the magnetic cations are randomly distributed over the five available *B*-sites in a perovskite block [3]. Electron microscopy [3] and density functional theory (DFT) [4] studies demonstrate that the inclusion of manganese within the B6TFMO

structure is crucial to promoting long-range ferrimagnetic order. Note that detailed micro- and nano-structural analysis, combined with rigorous statistics (confidence level ≥99.5%), conclude that ferrimagnetic/ferromagnetic secondary phase impurities do not influence the ferrimagnetic/ferromagnetic behavior [13].

3. Analysis of STEM-EELS spectra

Titanium is the most abundant *B*-site cation in B6TFMO, therefore the Ti $L_{2,3}$-edge is the most intense peak in the EELS spectra. There is a ~18 ± 4 % decrease in the amount of Ti at the center layers compared to the outer layers. The decrease in the Ti $L_{2,3}$-edge intensity from outer to center perovskite layer in the EELS data (**SI Figure S1 (a)**) is consistent with the decrease in Ti *B*-site occupancy observed in previous analysis by the HAADF-STEM EDX technique [3], confirming the preference for Ti to partition towards the outer layers in B6TFMO. Both Fe $L_{2,3}$-edge and Mn $L_{2,3}$-edge demonstrate a preference for the Fe and Mn cations to partition towards the intermediate and center layers, as shown in **SI Figure S1 (g)** and **(e)** by the increase in intensity from the outer layers towards the center layers of these edges. Although the signal-to-noise ratio was too low to reliably quantify the intensity changes, this observation confirms previous EDX observations of a significant increase in the *B*-site proportion of Mn[3] in the center layers, confirming a distinct preference for partitioning of magnetic Mn towards the center perovskite layers.

The O K-edge within B6TFMO provides electronic information on the bonding between O and its neighboring cations. Typically, in the O K-edge fine structure for metal oxides, much information can be gained about the local geometry of the complex [14,15]. The most intense component of the O K-edge in the data collected in **SI Figure S1 (c-d)**, which is not zero-loss aligned, is at an energy of ~522 eV and gives information on the unoccupied O 2*p* states, which hybridize with the *B*-site metal cation 3*d* states. First, we observe an energy splitting of ~2 eV in the O K-edge due to the weak hybridization between the transition metal cation 3*d* t_{2g} orbitals (lower peak) with the O 2*p* orbitals, and the more strongly hybridized 3*d* e_g orbitals (higher peak) with the O 2*p* orbitals. This splitting could also have a contribution from the interactions between the O 2*p* and Bi 5*d*/*p* states [16,17]. The second broad feature at ~532 eV in our spectra of the O K-

edge is attributed to the O 2*p* hybridization with the 4*s* and 4*p* states in the metal cations along with covalent bonding in Bi. Given that B6TFMO is a complex multi-cation oxide, it should be noted that the individual contributions of Ti, Mn and Fe to the O K-edge structure would be difficult to deconvolute. In our analysis, we did not differentiate any significant changes to the O K-edge structure in the EELS spectra as measured from the outer, intermediate or center perovskite layers and taking into account that B6TFMO is a complex multi-cation oxide, it would be difficult to deconvolute the individual contributions of Ti, Mn and Fe to the O K-edge structure. While peak splitting can be observed within the O K-edge as shown in **Figure S1 (c, d)**, the splitting between the t_{2g} and e_g peaks is not as well defined compared to the splitting within the Ti $L_{2,3}$-edge demonstrated in **SI Figure 1 (a-b)**. Accordingly, the Ti $L_{2,3}$-edge was selected for further investigation of the electronic structure and subtle chemical bonding changes through the B6TFMO structure, that is from the interface of the $[Bi_2O_2]^{2+}$-perovskite interface to the center of the perovskite block.

In previous works [1,3,4], we have demonstrated that the presence of Mn is key to the ferrimagnetic/ferromagnetic behavior observed within B6TFMO. EELS analysis in this work (**SI Figure S1 (e-f)**) confirms the previous EDX observations of the partitioning of Mn cations towards the center perovskite layers. Previous Density Functional Theory (DFT) calculations [4] indicate that magnetization values in B6TFMO are highly dependent on the increased nearest neighbor magnetic interactions resulting from this Mn partitioning. Considering the Goodenough−Kanamari rules, [18–20] ferromagnetic coupling could be created through Fe^{3+}–O–Mn^{4+} (d^5/d^3 configurations) super-exchange or Mn^{3+}–O–Mn^{4+} (d^4/d^3 configurations) double-exchange interactions. Ideally, EELS analysis would enable determination of the oxidation state of Mn within B6TFMO in order to determine the precise mechanism for ferromagnetic exchange [21,22]. However, due to the

comparatively low concentration of Mn (~1.7%) within B6TFMO's total composition, the Mn $L_{2,3}$-edge had a relatively low intensity compared to Ti, Fe and O signals within our data sets. The low signal to noise ratio within the Mn $L_{2,3}$-edge meant that the structural features characteristic of the different Mn oxidation states could not be observed, and it was not possible to determine the exact oxidation state of the Mn cation. The Fe cation oxidation state was confirmed by the presence of a shoulder at ~699 eV just after the edge onset in the L_3 signal. This initial feature is ~1 eV before the maximum peak, at ~700 eV, and the shape seen in **SI Figure S1 (g-h)** is characteristic of Fe^{3+} and is consistent with the literature [23–26].

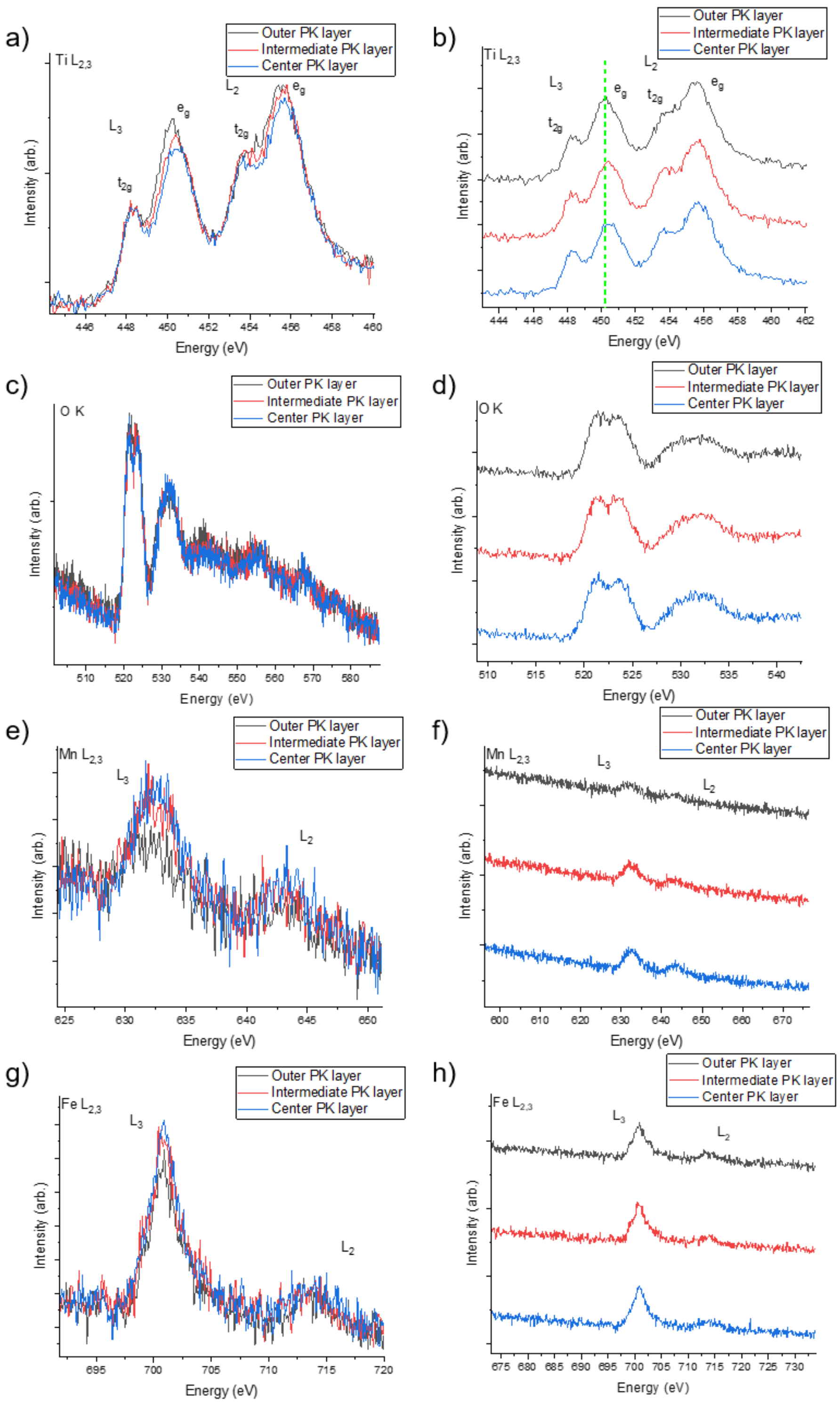

Figure S1. Atomic resolution STEM EELS spectra of outer, intermediate, and center perovskite regions of five-layered B6TFMO. **a)** and **b)** Ti $L_{2,3}$-edge, **c)** and **d)** O K-edge, **e)** and **f)** Mn $L_{2,3}$-edge, **g)** and **h)** Fe $L_{2,3}$-edge.

Data Set Label	Outer Ti4+ L3 eg Peak (eV)	Centre Ti4+ L3 eg Peak (eV)	Change in Δ (eV)
210610 K2 11 i	453.77	453.87	0.1
210610 K2 11 ii	453.8	453.87	0.07
210520 K2 7 i	450.24	450.44	0.2
210520 K2 7 ii	450.36	450.44	0.08
210520 K2 8 i	450.65	450.88	0.23
210520 K2 8 ii	450.61	450.88	0.27
210520 K2 12 i	458.06	458.15	0.09
210520 K2 12 ii	458.06	458.15	0.09
211002 K2 12 i	458.44	458.51	0.07
211002 K2 12 ii	458.33	458.51	0.18
211002 K2 16 i	458.75	458.83	0.08
211002 K2 16 ii	458.71	458.83	0.12
211201 K2 2.5 32 i	458.35	458.38	0.03
211201 K2 2.5 32 ii	458.26	458.38	0.12
211201 K2 5 29 i	457.86	458.00	0.14
211201 K2 5 29 ii	457.83	458.00	0.17
211201 K2 5 31 i	458.14	458.24	0.1
211201 K2 5 31 ii	458.14	458.24	0.1
US100 2.5 13 A i	460.12	460.26	0.14
US100 2.5 13 A ii	460.04	460.26	0.22
US100 2.5 13 B i	460.2	460.28	0.08
US100 2.5 13 B ii	460.14	460.28	0.14
		Average	0.13 eV
		St. Dev	0.06 eV

Table S1. Data from which the average change in crystal field splitting (Δ) for the Ti^{4+} L_3 edge is calculated. Eleven EELS data sets were used to calculate the average Δ value. Each data set had two e_g values for the outer perovskite layer and one e_g value for the center layer, therefore for each data set, two Δ values were obtained (twenty-two data sets were obtained in total). The t_{2g} peak value remains the same for the EELS spectra moving from the outer to the center perovskite layers while the e_g peak value changes. The change in Δ moving from the outer to the center perovskite layers for the Ti^{4+} L_3 peak was calculated by subtracting the outer perovskite layer Ti^{4+} L_3 e_g peak value from the center perovskite Ti^{4+} L_3 e_g layer value.

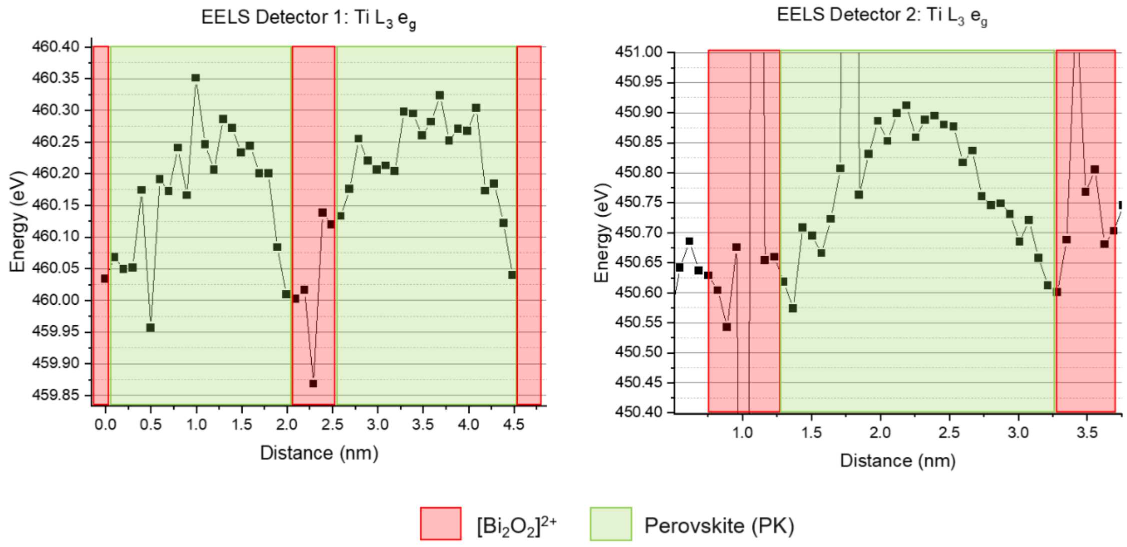

Figure S2. Further examples of plots from EELS data sets to support **Figure 2 (d)** in the main text, where Detector 1 is the US1000 and Detector 2 is the K2. These plots demonstrate the change in peak energy of the Ti L_3 e_g peak from re-binned EELS spectra as a function of distance through the perovskite layers of B6TFMO.

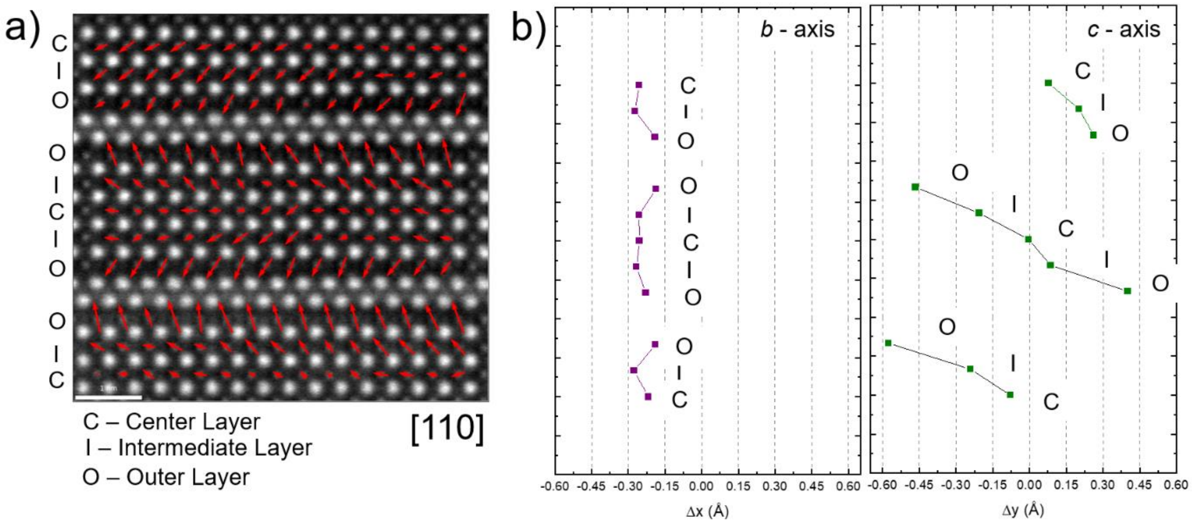

Figure S3. a) STEM-HAADF image of five-layered B6TFMO overlaid with polarization vector mapping represented by the red arrows. Once the *A* and *B* site positions of the atoms are determined, the polarization vectors (red) were obtained from the reverse of the *B*-site cation displacements using TEMUL toolkit [27]. The average *B*-site displacement within a perovskite cell for outer, intermediate and center *B*-sites were calculated along the *b*-axis (Δx) and *c*-axis (Δy) direction and are plotted in **b)**. The overall average magnitude from each type of perovskite layer (outer, intermediate, center) for the *B*-site cations displacements in the *c*-axis (Δy) direction were outer: 0.426 ± 0.142 Å, intermediate: 0.184 ± 0.095 Å and center: 0.054 ± 0.062. Average values for *B*-site cation displacements along the *b*-axis (Δx) were outer: 0.199 ± 0.006 Å, intermediate: 0.268 ± 0.009 Å and center: 0.243 ± 0.009 Å.

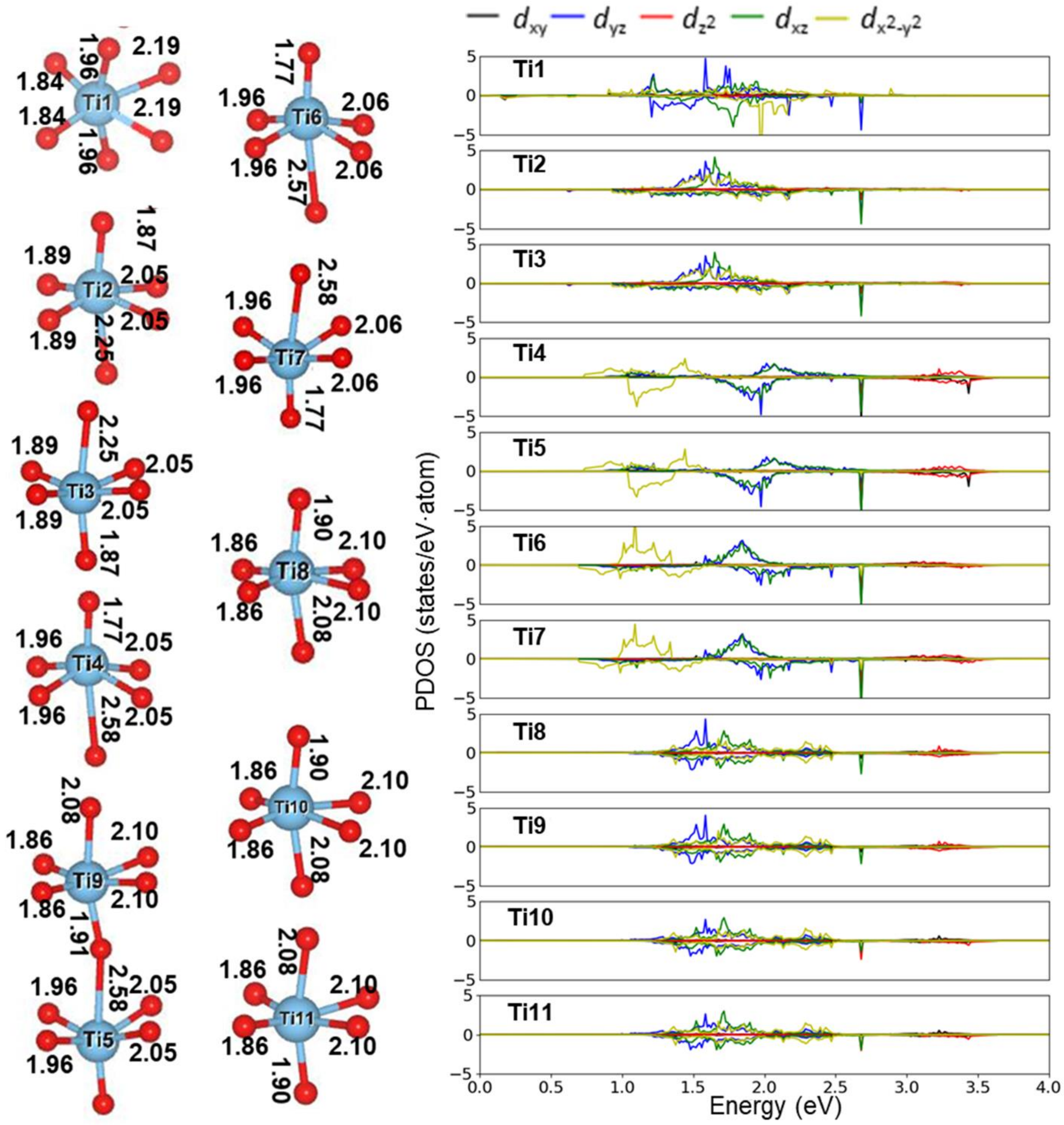

Figure S4. This is supplementary information to **Figure 3** in the main text (*Configuration*: *a*). All of the Ti-O bond lengths in Å and PDOS calculations for all of the Ti atoms of the $Bi_{24}Ti_{11}Fe_6Mn_3O_{72}$ configuration are shown.

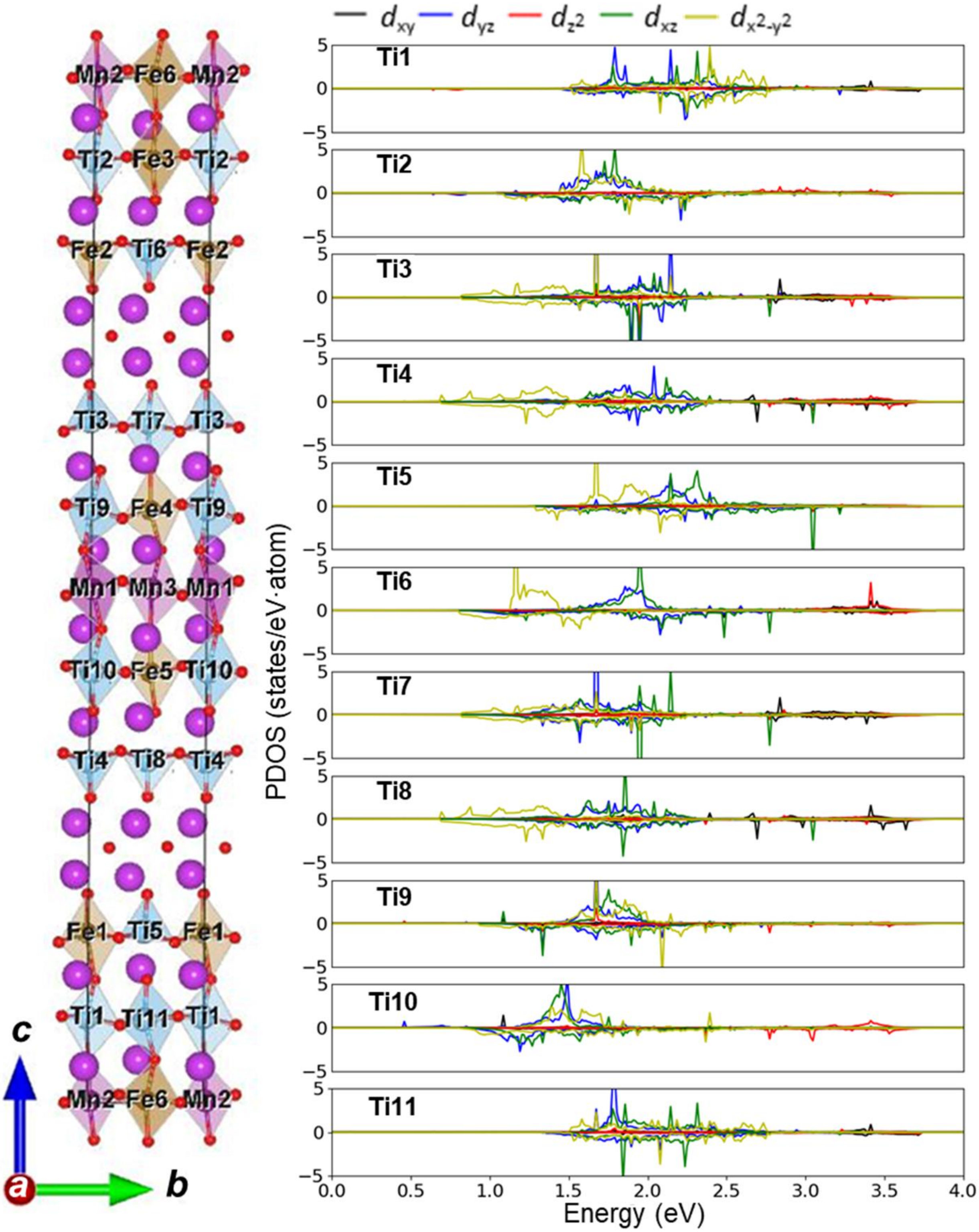

Figure S5. We consider another configuration (*Configuration*: *b*) of atoms and magnetic moments

with differing locations for the *B*-site cations to that shown for the configuration (*Configuration*: *a*) in **Figure 3**/**SI Figure S4**. The fully optimized structure (both lattice parameters and atomic positions) calculated for this configuration of $Bi_{24}Ti_{11}Fe_6Mn_3O_{72}$ is shown. Our calculated lattice parameters for *a*, *b*, and *c* are 5.53 Å, 5.63 Å, and 49.93 Å, respectively. Similar to the previous configuration, we observe that Ti/Fe/Mn atoms are displaced along the negative *b*-axis, producing a dipole moment, thus net ferroelectric polarization lies along the *b*-axis. We also notice that Ti/Fe/Mn atoms move along the *c*-direction upward and downward (significant displacement of the Outer Ti atoms) in an antipolar arrangement toward the $[Bi_2O_2]^{2+}$ layers, which would result in a net zero dipole along the *c*-direction if the horizontal mirror plane was allowed to persist. (Right) The atom resolved PDOS are shown, where positive (negative) PDOS represents up (down) spin channels. The Fermi energy (E_F) is set at 0 eV. The configuration is in a ferrimagnetic state with net magnetic moment of 33.892 μ_B per unit cell, where Fe1, Fe2, Fe3, Fe4, Fe5, Fe6, Mn1, Mn2, and Mn3 atoms contribute 4.341 μ_B, 4.322 μ_B, 4.365 μ_B, 4.351 μ_B, 4.348 μ_B, 4.306 μ_B, 3.596 μ_B, -3.348 μ_B, 3.809 μ_B, respectively. Positive (negative) signs of the magnetic moment represent up (down) spin states. Other atoms contribute very little to the overall magnetic moment. Note that *Configuration*: *b* is slightly (0.18 meV/atom) lower in energy relative to *Configuration*: *a*. In fact, there are many possible atomic and magnetic configurations for Mn and Fe atoms to be placed in real samples. While we present two possible configurations within this study, we propose that the antipolar Ti displacement along *c*-direction within each perovskite block will be observed irrespective of the atomic and magnetic configurations of Mn and Fe atoms.

4. Octahedral tilting in Aurivillius phase systems

It has been proposed [28,29] that symmetry lowering in the Aurivillius phases is caused by tilting of the oxygen octahedra around the *a*-axis (tilt mode designated by the irreducible representation (*irrep*) notation X_3^+), rotations of the oxygen octahedra around the *c*-axis (i*rreps* X_2^+, X_1^-), (shown in **SI Figure S6**), and the polar cation motions along the *a*-axis (*irrep* Γ_5^-), which when coupled together contribute to a material's ferroelectric ground state. Goldschmidt's tolerance factor [30], $t = \frac{r_o + r_a}{\sqrt{2}(r_o + r_b)}$, where r_o, r_a and r_b are the ionic radii of the oxygen, *A*-site and *B*-site ions respectively, has been used to deduce that $t < 1$ would promote a perovskite system with an instability towards an octahedral tilting distortion [5,31,32]. This tolerance factor can also be applied to the Aurivillius phases, where octahedral tilting is commonly observed [5,7,10,28,33] due to divergences between the *A*-O and *B*-O interatomic distances.

Research on the *m* = 3 phase, $Bi_4Ti_3O_{12}$ (BTO), has shown the importance of analyzing the contributions of the light oxygen atoms to denoting the symmetry and space group of the material. In the 1990s it was proposed that above the Curie temperature (T_c) ~675 °C, BTO adopts the aristotype *I*4/*mmm* structure, while at RT a subgroup of *Fmmm*: *B*1*a*1 is present [34–36]. However, theoretical calculations found no triggering mechanism to support a single transition from the *I*4/*mmm* to *B*1*a*1 space group [29], leading researchers to revisit the BTO system in 2019 [7], where an intermediate paraelectric phase above T_c was discovered corresponding to the irrep X_2^+ mode, *P*4/*mbm* (**SI Figure S6**). The BTO case highlights the significance of the oxygen octahedral tilting on the resultant symmetry and polar properties of the Aurivillius structure and demonstrates that visualization of the light atoms is critical for correct structural characterization of a material. It has also been found for the higher phase *m* = 4 systems [33], $Bi_5Ti_3FeO_{15}$ and $SrBi_4Ti_4O_{15}$ that at RT the *A*2$_1$*am* space group is present, which allows movement of the oxygen atoms and BO_6 octahedral

tilting, rather than the once proposed *Fmm*2 space group [37]. Analysis on the m = 3 and m = 4 systems shows the advantages neutron diffraction has over X-ray diffraction, as in these systems the additional symmetry lowering arises from displacements of the oxygen atoms via octahedral tilting, which do not give rise to strong reflections within X-ray diffraction patterns.

Of the five-layered Aurivillius phases that have been investigated, one of the most compositionally similar materials to B6TFMO is $Bi_6Ti_3Fe_2O_{18}$, where García-Guaderrama *et al.* [38] deduced the space group to be *F*2*mm* via synchrotron X-ray powder diffraction, which did not detect tilting of the TiO_6 octahedra. However, samples of the five layered Aurivillius system $A_2Bi_4Ti_5O_{18}$, substituted by Ca, Sr, Pb or Ba at the *A*-sites, were refined to the orthorhombic *B2eb* space group in a neutron diffraction study by Ismunandar *et al.* [5]. The tolerance factors were calculated to be 0.97, 1.0, 1.02 and 1.06 for Ca, Sr, Pb or Ba substituted structures, respectively, with an increase in the degree of octahedral tilting corresponding with a decrease in the tolerance factor, as shown in Table S2 of the SI. Among the compositions within this m = 5 system previously characterized [5] by neutron diffraction, $Ca_2Bi_4Ti_3O_{18}$ displays the highest orthorhombic distortion (t = 0.97), and exhibits tilt angles of ±11 ° for center, ±10 ° for intermediate and ±7 ° for outer perovskite octahedra, as illustrated in **SI Table S2**.

The calculation of the Goldschmidt tolerance factor [30] for B6TFMO results in a value of 0.95. The average radius of the *B*-site ions (r_b) was determined using a 3:1.5:0.5 ratio for Ti^{4+}:Fe^{3+}:Mn^{3+}. This tolerance factor of < 1 indicates that octahedral tilting within B6TFMO is favorable.

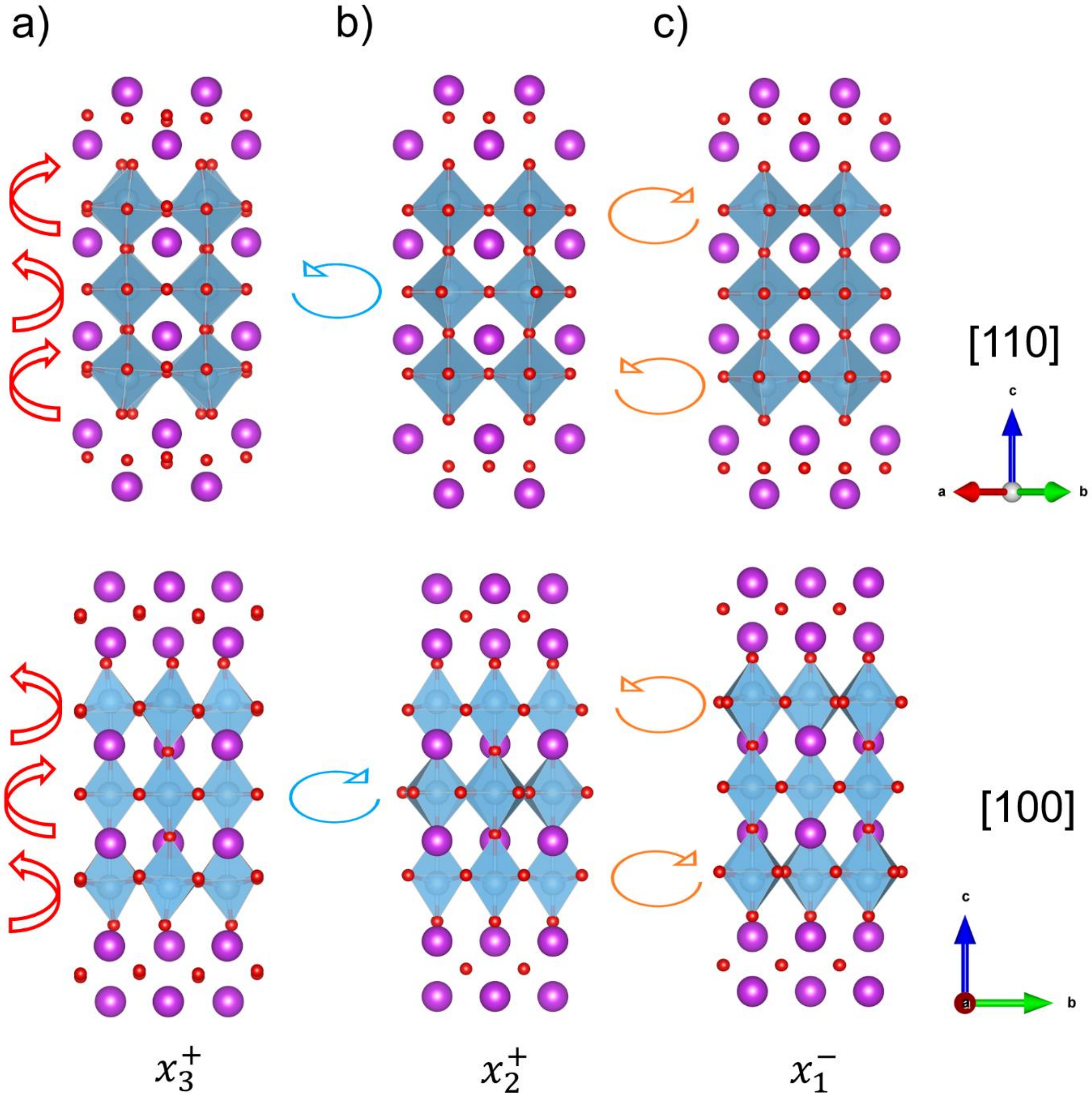

Figure S6. Illustration of individual tilt modes found in three-layered Aurivillius phases. Structures from Guo *et al*. [7] are used to visualize these octahedral tilt modes. **a)** *P*4*/ncm* structure demonstrating the anti-phase tilting in the *ab* plane, associated with the irrep X_3^+ mode. **b)** Cmce space group, where the irrep X_2^+mode provides a rotation around *c* at the center perovskite layer. **c)** *P*4*/nbm* space group, where the irrep X_1^-mode has an anti-phase rotation around *c*, in the outer perovskite layers next to $[Bi_2O_2]^{2+}$.

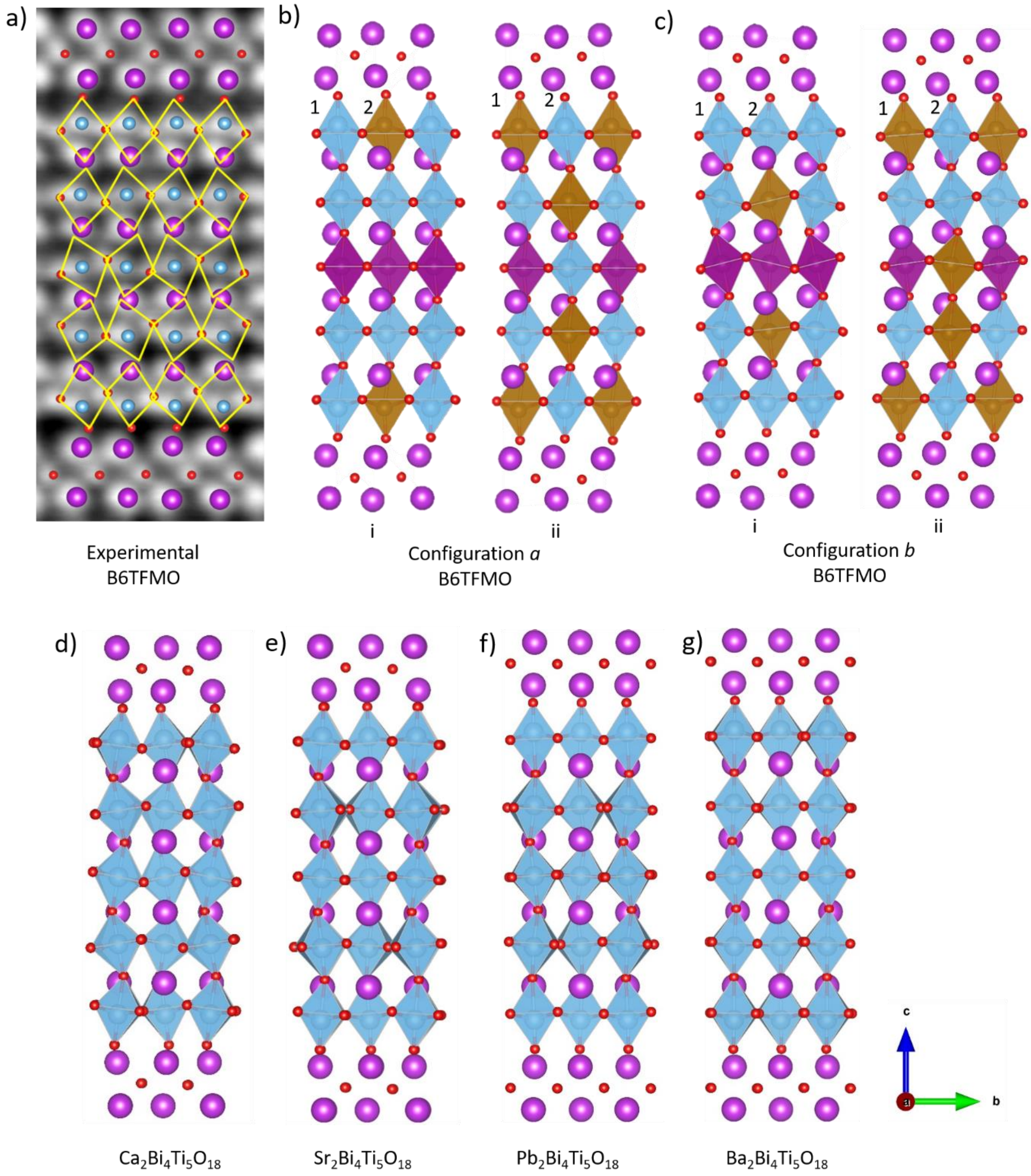

Figure S7. Projected images down [100] used to approximate tilt angles associated with anti-phase tilting of perovskite octahedra, correlated with the irrep X_3^+ mode. Given that positions of the perovskite *A*-site bismuth atoms coincide with positions of apex oxygen atoms, accurate O-*B*-O (*B*-site to oxygen) bond angles could not be determined from the 2*D* iDPC images. To approximate

tilt angles for the perovskite octahedra, a 2*D* diamond shape (yellow) shown in **(a)** was created to match the positions determined for the apex and equatorial oxygen atoms of the BO_6 octahedra (when viewed down the [100] projection). To allow a comparison of tilt angles from experimental and theoretical data, the yellow diamond was overlaid onto iDPC images **(a)**, atomistic structures simulated by DFT (**(b)**, **(c)**) and atomistic structures determined from neutron diffraction data from literature (**(d)** to **(g)**) [5]. One Aurivillius unit cell is comprised of two five-layered perovskite blocks. To enable the desired $Bi_{24}Ti_{11}Fe_6Mn_3O_{72}$ stoichiometry, two different half-unit cell arrangements of the Ti, Fe and Mn cations are required for the DFT structures simulated in **(b)** and **(c)**. These two different arrangements are represented by the half-unit cell blocks labelled **(i)** and **(ii)**. The atomic arrangement of *Configuration*: *b* is such that not all the outer and intermediate perovskite cells have the same arrangement of *B*-site cations. See **Figure S4** and **Figure S5** for the whole unit cell and **Figure S8** and **Figure S9** for the bond lengths, angles, and atomic displacement along the *c*-axis direction. A manual fit to the underlying octahedra was applied using the position of the oxygen atoms as a guide. The yellow diamond is considered to be at a 0° tilt angle when equatorial oxygen atoms lie along the same horizontal plane. Octahedral tilting results in deviations from 0° and values determined for the outer, intermediate and center perovskite layers are displayed in **SI Table S2**.

Layer	Tilt (°)				
	Experimental B6TFMO [100]	$Ca_2Bi_4Ti_3O_{18}$ [100]	$Sr_2Bi_4Ti_3O_{18}$ [100]	$Pb_2Bi_4Ti_3O_{18}$ [100]	$Ba_2Bi_4Ti_3O_{18}$ [100]
Outer	±5	±7	±4	±2	±1
Intermediate	±11	±10	±5	±4	±2
Center	±16	±11	±4	±6	±2

Layer	DFT Configuration: *a*				DFT Configuration: *b*			
	B6TFMO [100] i		B6TFMO [100] ii		B6TFMO [100] i		B6TFMO [100] ii	
	Column 1	Column 2	Column 1	Column 2	Column 1	Column 2	Column 1	Column 2
Outer (top)	-4	-2	-3	-4	-6	2	-5	1
Intermediate (top)	-1	-1	-2	-2	13	-11	8	-8
Center	0	0	1	1	-14	11	-6	6
Intermediate (bottom)	1	1	2	2	6	-4	4	1
Outer (bottom)	4	2	3	4	2	6	3	3

Table S2. Octahedral tilt angles along the <010> direction for experimental B6TFMO are approximated from iDPC images in **Figure S7 (a)**, and compared to structures of five-layered Aurivillius phases $A_2Bi_4Ti_5O_{18}$ determined from literature neutron diffraction data [5] (**Figure S7 (d-g))** and DFT simulated configurations **(Figure S7 (b-c))**. This table shows the degree of tilt for the BO_6 octahedra away from 0° for the *irrep* X_3^+ tilt mode (in-plane (perpendicular to *c*) anti-phase tilting of the octahedra with respect to each other in the *ab*-plane). Values for the experimental B6TFMO [100] are averaged as measured from **Figure S7 (a)** which can be also seen in **Figure 5 (c)** of the main text. The average was calculated from the four columns as observed in **Figure S7 (a)**, where the value for the outer and intermediate layers were each averaged over eight values (four from the top layer and four from the bottom layer). In this table, a minus value (–) indicates a tilt towards the left while a plus value (+) indicates a tilt towards the right. Except for **Figure S7 (b)** and **(c)** (Configuration: *a* and *Configuration*: *b*), the tilting direction alternates between right and left (+/-) for the structures shown in Figure S7 as one moves through the perovskite layer in both the *c*-axis and *b*-axis direction. The DFT configurations which include Mn, Fe and Ti at the *B*-sites are more complex than the neutron diffraction crystallographic file structures from Ismunander *et al.* [5] (having only Ti at the *B*-sites) and have two distinct columns (marked 1 and 2 in **Figure S7 (b)-(c)**), where the tilt angles differ due to the differing arrangement of the *B*-site

cations within the Aurivillius half-unit cell. DFT *Configuration*: *a* and the neutron diffraction data have perovskite blocks where there are only three unique layers across the five perovskite sites. The two outer layers (top and bottom) have an equivalent arrangement of *B*-cations and the two intermediate layers (top and bottom) have an equivalent arrangement of *B*-cations. Therefore, the two outer and two intermediate octahedra in the five-layered perovskite block have the same degree of tilt, but with differing direction, as indicated by the +/- sign. In DFT *Configuration*: *b* there are five unique layers in the perovskite block at the center of the unit cell (**Figure S7 (c) ii**, **S8** and **S9**). The two outer layers (top and bottom) are not equivalent nor are the two intermediate layers (top and bottom), therefore five tilt values are obtained for this structure.

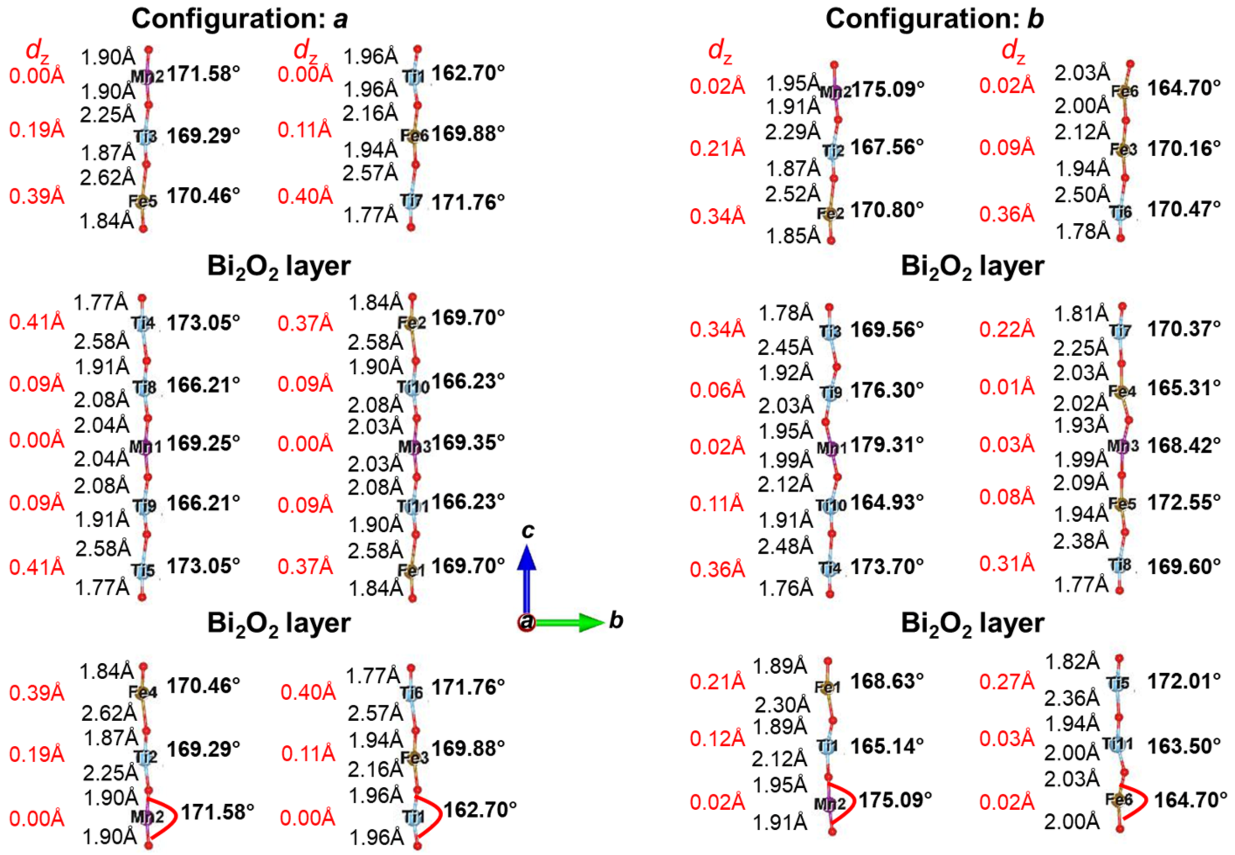

Figure S8. The positions of all oxygen and *B*-site atoms can be accurately measured from the DFT simulated structures. This figure displays bond angles at each *B*-site cation (Ti/Fe/Mn) between two *B*-O bonds along the *c*-direction for both our calculated atomic configurations. In general, we see that the bond angle deviates from 180° due to displacement of Ti/Fe/Mn atoms as well as octahedral tilting along the *a*-direction. The Ti/Fe/Mn-O bond lengths are shown. As discussed in the main text, we observe short (l_s) and long (l_l) bonds along the *c*-direction as we go from the center layer to the outer layer. We define the displacement (d_z) of Ti/Fe/Mn along the *c*-direction as $(l_l - l_s)/2$, shown in red text in the figure. In agreement with our experimental STEM data, we notice that d_z of Ti/Fe/Mn increases as we go from the center layer to the outer layer.

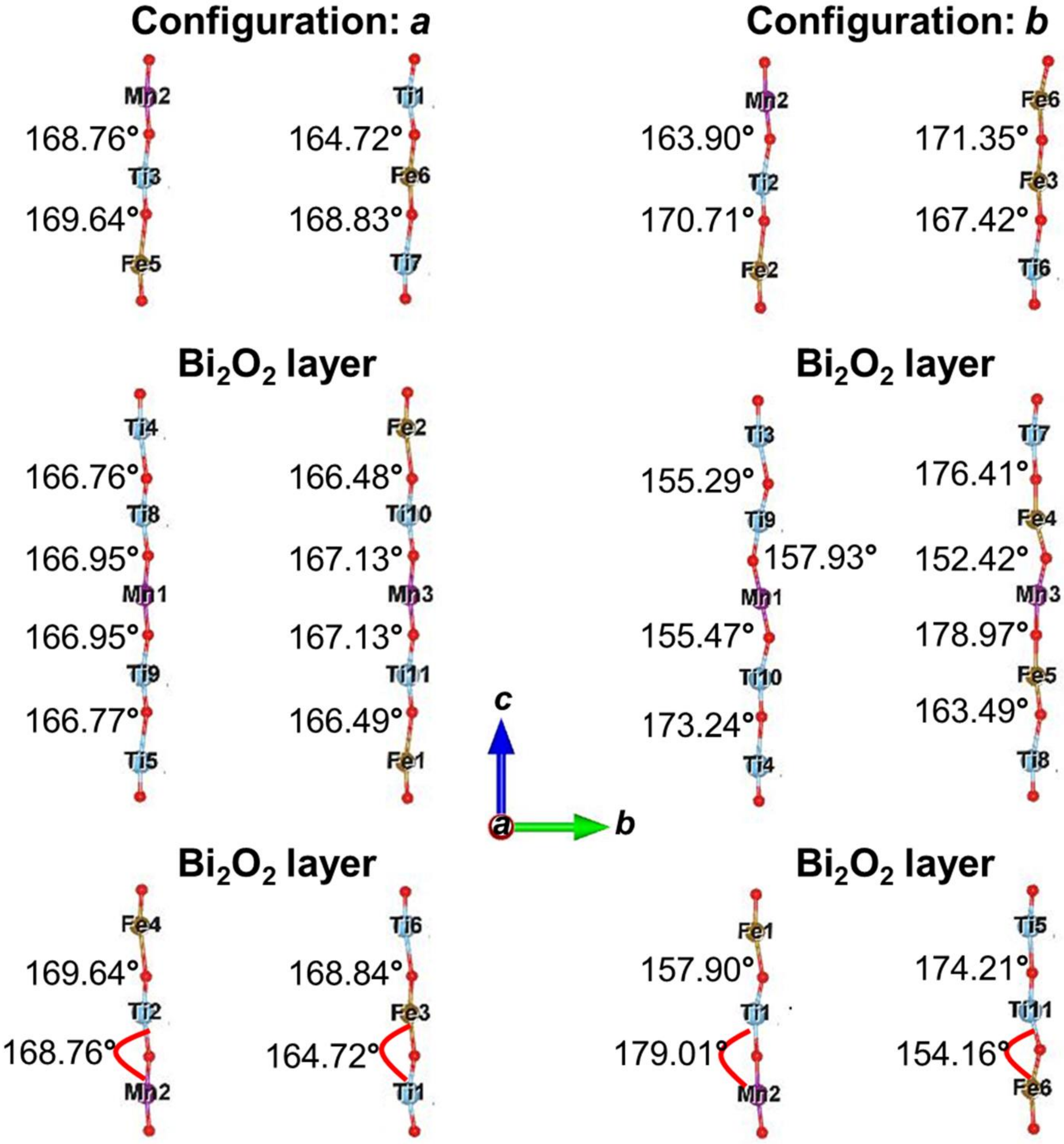

Figure S9. The positions of all oxygen and *B*-site atoms can be accurately measured from the DFT simulated structures. This figure displays bond angles at each oxygen (O)-site between two *B*-O bonds along the *c*-direction for both our calculated atomic configuration. Without any distortion or tilting, the angle should be 180°. Overall, the angles deviate from 180° significantly (by 12.87 ° to 27.58 °) along the *b*-direction at the center layer. Note that *Configuration*: *a* and *Configuration*:

b have different atomic arrangements of Ti/Fe/Mn atoms, which results in different magnitudes for the tilt angles.

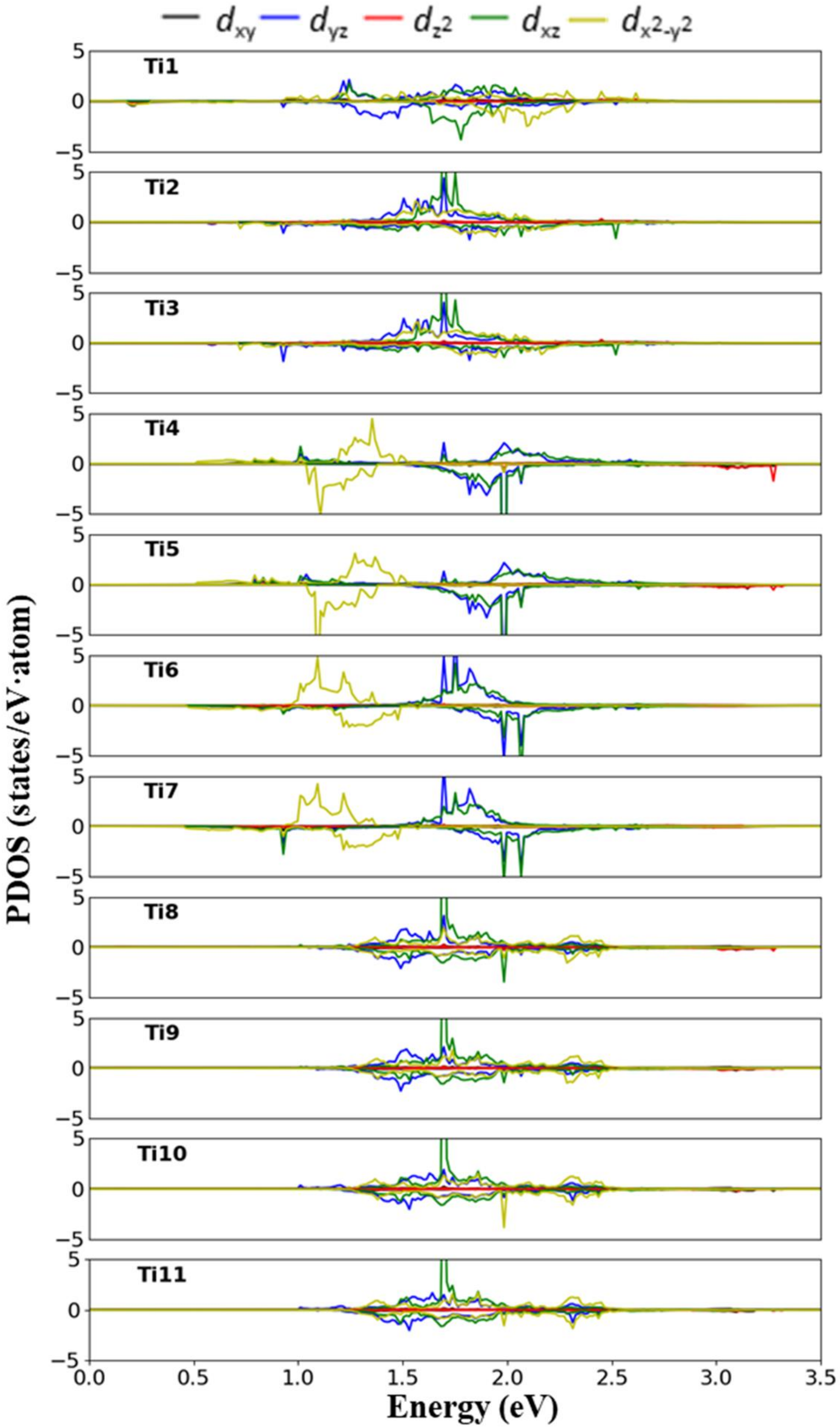

d_{xy} d_{yz} d_{z^2} d_{xz} $d_{x^2-y^2}$
Ti1
Ti2
Ti3
Ti4
Ti5
Ti6
Ti7
Ti8
Ti9
Ti10
Ti11
PDOS (states/eV·atom)
Energy (eV)
5
0
−5
0.0
0.5
1.0
1.5
2.0
2.5
3.0
3.5

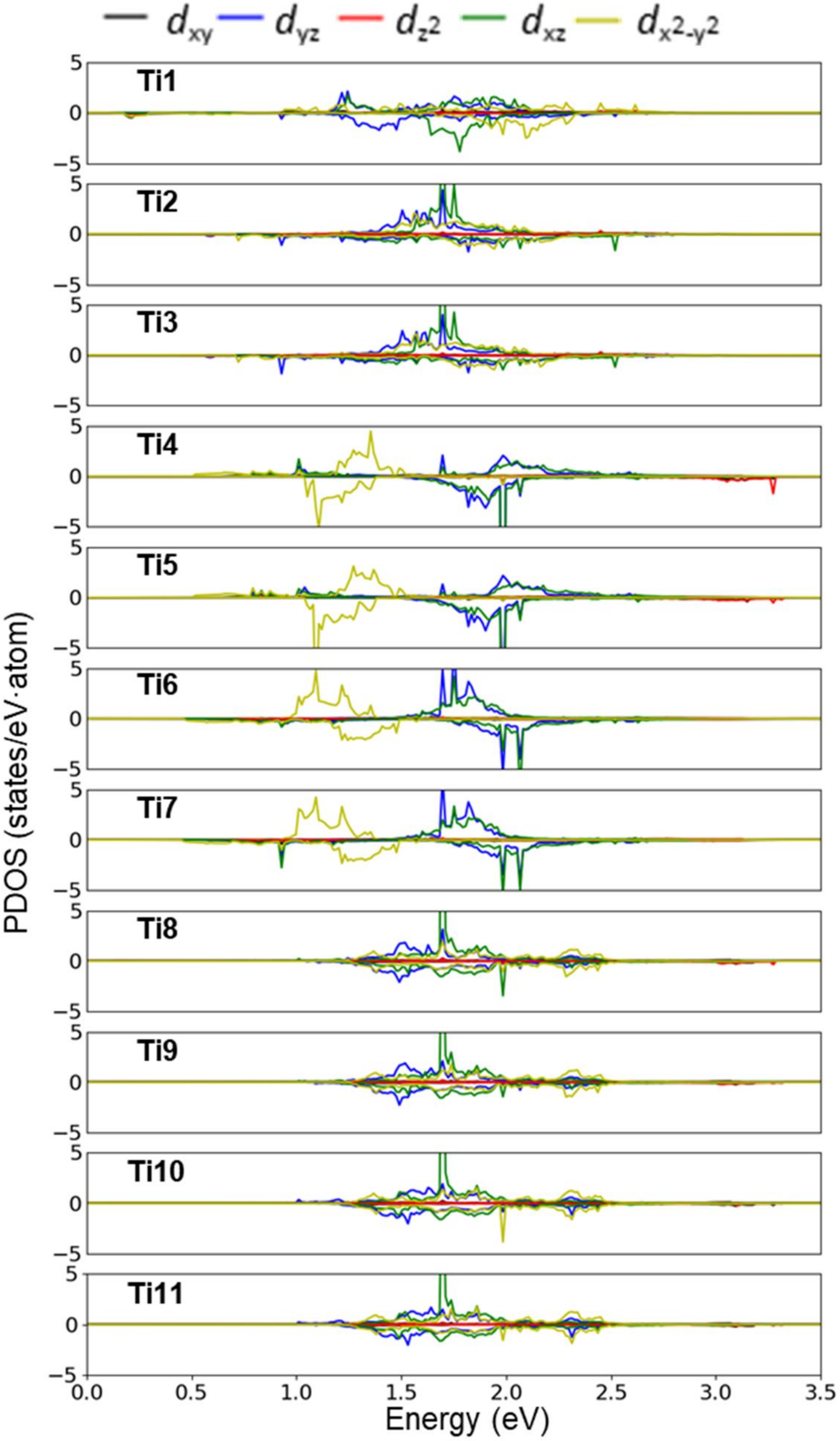

d_{xy} d_{yz} d_{z^2} d_{xz} $d_{x^2-y^2}$
Ti1
Ti2
Ti3
Ti4
Ti5
Ti6
Ti7
Ti8
Ti9
Ti10
Ti11
PDOS (states/eV·atom)
Energy (eV)
5
0
−5
0.0
0.5
1.0
1.5
2.0
2.5
3.0
3.5

Figure S10. For *Configuration*: *a*, we also consider U values of 4.0 eV and 3.0 eV for Fe and Mn, respectively. Our calculated lattice parameters for *a*, *b*, and *c* are 5.48 Å, 5.61 Å, and 51.08 Å, respectively. These values align with those reported in the main text (where *a*, *b*, and *c* are 5.48 Å, 5.61 Å, and 51.03 Å, respectively) for U = 5.5 eV for Fe and U = 3.0 eV for Mn. Notably, there is no significant alteration in the structure observed with variation of U values. Here, the atom resolved PDOS for Ti atoms are presented, where positive (negative) PDOS represents up (down) spin channels. The Fermi energy (E_F) is set at 0 eV. Upon comparison with Figure S4, it is evident that adjustments in U values do not impact the overall conclusions drawn in this manuscript.

REFERENCES

(1) Keeney, L.; Maity, T.; Schmidt, M.; Amann, A.; Deepak, N.; Petkov, N.; Roy, S.; Pemble, M. E.; Whatmore, R. W. Magnetic Field-Induced Ferroelectric Switching in Multiferroic Aurivillius Phase Thin Films at Room Temperature. *J. Am. Ceram. Soc.* **2013**, *96* (8), 2339–2357. https://doi.org/10.1111/jace.12467.
(2) Faraz, A.; Maity, T.; Schmidt, M.; Deepak, N.; Roy, S.; Pemble, M. E.; Whatmore, R. W.; Keeney, L. Direct Visualization of Magnetic-Field-Induced Magnetoelectric Switching in Multiferroic Aurivillius Phase Thin Films. *J. Am. Ceram. Soc.* **2017**, *100* (3), 975–987. https://doi.org/10.1111/jace.14597.
(3) Keeney, L.; Downing, C.; Schmidt, M.; Pemble, M. E.; Nicolosi, V.; Whatmore, R. W. Direct Atomic Scale Determination of Magnetic Ion Partition in a Room Temperature Multiferroic Material. *Sci. Rep.* **2017**, *7* (1), 1737. https://doi.org/10.1038/s41598-017-01902-1.
(4) Moore, K.; O'Connell, E. N.; Griffin, S. M.; Downing, C.; Colfer, L.; Schmidt, M.; Nicolosi, V.; Bangert, U.; Keeney, L.; Conroy, M. Charged Domain Wall and Polar Vortex Topologies in a Room-Temperature Magnetoelectric Multiferroic Thin Film. *ACS Appl. Mater. Interfaces* **2022**, *14* (4), 5525–5536. https://doi.org/10.1021/acsami.1c17383.
(5) Ismunandar; Kamiyama, T.; Hoshikawa, A.; Zhou, Q.; Kennedy, B. J.; Kubota, Y.; Kato, K. Structural Studies of Five Layer Aurivillius Oxides: $A_2Bi_4Ti_5O_{18}$ (A=Ca, Sr, Ba and Pb). *J. Solid State Chem.* **2004**, *177* (11), 4188–4196. https://doi.org/10.1016/j.jssc.2004.07.032.
(6) Newnham, R. E.; Wolfe, R. W.; Dorrian, J. F. Structural Basis of Ferroelectricity in the Bismuth Titanate Family. *Mater. Res. Bull.* **1971**, *6* (10), 1029–1039. https://doi.org/10.1016/0025-5408(71)90082-1.
(7) Guo, Y.-Y.; Gibbs, A. S.; Perez-Mato, J. M.; Lightfoot, P. Unexpected Phase Transition Sequence in the Ferroelectric $Bi_4Ti_3\ O_{12}$. *IUCrJ* **2019**, *6* (3), 438–446. https://doi.org/10.1107/S2052252519003804.
(8) Armstrong, R. A.; Newnham, R. E. Bismuth Titanate Solid Solutions. *Mater. Res. Bull.* **1972**, *7* (10), 1025–1034. https://doi.org/10.1016/0025-5408(72)90154-7.

(9) Kikuchi, T. Stability of Layered Bismuth Compounds in Relation to the Structural Mismatch. *Mater. Res. Bull.* **1979**, *14* (12), 1561–1569. https://doi.org/10.1016/0025-5408(72)90226-7.
(10) Giddings, A. T.; Stennett, M. C.; Reid, D. P.; McCabe, E. E.; Greaves, C.; Hyatt, N. C. Synthesis, Structure and Characterisation of the N=4 Aurivillius Phase $Bi_5Ti_3CrO_{15}$. *J. Solid State Chem.* **2011**, *184* (2), 252–263. https://doi.org/10.1016/j.jssc.2010.09.031.
(11) Lomanova, N.; Semenov, V.; Panchuk, V.; Gusarov, V. Structural Changes in the Homologous Series of the Aurivillius Phases $Bi_{n+1}Fe_{n-3}Ti_3O_{3n+3}$. *J. Alloys Compd.* **2012**, *528*, 103–108. https://doi.org/10.1016/j.jallcom.2012.03.040.
(12) Shannon, R. D. Revised Effective Ionic Radii and Systematic Studies of Interatomic Distances in Halides and Chalcogenides. *Acta Crystallogr. Sect. A* **1976**, *32* (5), 751–767. https://doi.org/10.1107/S0567739476001551.
(13) Schmidt, M.; Amann, A.; Keeney, L.; Pemble, M. E.; Holmes, J. D.; Petkov, N.; Whatmore, R. W. Absence of Evidence ≠ Evidence of Absence: Statistical Analysis of Inclusions in Multiferroic Thin Films. *Sci. Rep.* **2014**, *4* (1), 5712. https://doi.org/10.1038/srep05712.
(14) de Groot, F. M. F.; Grioni, M.; Fuggle, J. C.; Ghijsen, J.; Sawatzky, G. A.; Petersen, H. Oxygen 1s X-Ray-Absorption Edges of Transition-Metal Oxides. *Phys Rev B* **1989**, *40* (8), 5715–5723. https://doi.org/10.1103/PhysRevB.40.5715.
(15) Kourkoutis, L. F.; Xin, H. L.; Higuchi, T.; Hotta, Y.; Lee, J.-H.; Hikita, Y.; Schlom, D. G.; Hwang, H.; Muller, D. A. Atomic-Resolution Spectroscopic Imaging of Oxide Interfaces. *Philos. Mag.* **2010**, *90*, 4731–4749.
(16) Park, T.-J.; Sambasivan, S.; Fischer, D. A.; Yoon, W.-S.; Misewich, J. A.; Wong, S. S. Electronic Structure and Chemistry of Iron-Based Metal Oxide Nanostructured Materials: A NEXAFS Investigation of $BiFeO_3$, $Bi_2Fe_4O_9$, α-Fe_2O_3, γ-Fe_2O_3, and Fe/Fe_3O_4. *J. Phys. Chem. C* **2008**, *112* (28), 10359–10369. https://doi.org/10.1021/jp801449p.
(17) Sæterli, R.; Selbach, S. M.; Ravindran, P.; Grande, T.; Holmestad, R. Electronic Structure of Multiferroic $BiFeO_3$ and Related Compounds: Electron Energy Loss Spectroscopy and Density Functional Study. *Phys Rev B* **2010**, *82* (6), 064102. https://doi.org/10.1103/PhysRevB.82.064102.
(18) Goodenough, J. B. Theory of the Role of Covalence in the Perovskite-Type Manganites [La, M(II)]MnO_3. *Phys Rev* **1955**, *100* (2), 564–573. https://doi.org/10.1103/PhysRev.100.564.
(19) Goodenough, J. B. *Magnetism and the Chemical Bond*; Interscience publishers, 1963; Vol. 1.
(20) Goodenough, J. B. Jahn-Teller Phenomena in Solids. *Annu. Rev. Mater. Sci.* **1998**, *28* (1), 1–27. https://doi.org/10.1146/annurev.matsci.28.1.1.
(21) Gilbert, B.; Frazer, B. H.; Belz, A.; Conrad, P. G.; Nealson, K. H.; Haskel, D.; Lang, J. C.; Srajer, G.; De Stasio, G. Multiple Scattering Calculations of Bonding and X-Ray Absorption Spectroscopy of Manganese Oxides. *J. Phys. Chem. A* **2003**, *107* (16), 2839–2847. https://doi.org/10.1021/jp021493s.
(22) Garvie, L. A. J.; Craven, A. J. High-Resolution Parallel Electron Energy-Loss Spectroscopy of Mn $L_{2,3}$-Edges in Inorganic Manganese Compounds. *Phys. Chem. Miner.* **1994**, *21* (4), 191–206. https://doi.org/10.1007/BF00202132.
(23) Tan, H.; Verbeeck, J.; Abakumov, A.; Tendeloo, G. V. Oxidation State and Chemical Shift Investigation in Transition Metal Oxides by EELS. *Ultramicroscopy* **2012**, *116*, 24–33. https://doi.org/10.1016/j.ultramic.2012.03.002.
(24) Garvie, L. A. J.; Buseck, P. R. Ratios of Ferrous to Ferric Iron from Nanometre-Sized Areas in Minerals. *Nature* **1998**, *396* (6712), 667–670. https://doi.org/10.1038/25334.

(25) Colliex, C.; Manoubi, T.; Ortiz, C. Electron-Energy-Loss-Spectroscopy near-Edge Fine Structures in the Iron-Oxygen System. *Phys Rev B* **1991**, *44* (20), 11402–11411. https://doi.org/10.1103/PhysRevB.44.11402.
(26) Laan, G. van der; Kirkman, I. W. The 2p Absorption Spectra of 3d Transition Metal Compounds in Tetrahedral and Octahedral Symmetry. *J. Phys. Condens. Matter* **1992**, *4* (16), 4189. https://doi.org/10.1088/0953-8984/4/16/019.
(27) O'Connell, E.; Hennessy, M.; Moynihan, E. *PinkShnack/TEMUL:Zenodo*.
(28) Benedek, N. A.; Rondinelli, J. M.; Djani, H.; Ghosez, P.; Lightfoot, P. Understanding Ferroelectricity in Layered Perovskites: New Ideas and Insights from Theory and Experiments. *Dalton Trans* **2015**, *44* (23), 10543–10558. https://doi.org/10.1039/C5DT00010F.
(29) Perez-Mato, J. M.; Blaha, P.; Schwarz, K.; Aroyo, M. I.; Orobengoa, D.; Etxebarria, I.; García, A. Multiple Instabilities in $Bi_4Ti_3O_{12}$: A Ferroelectric beyond the Soft-Mode Paradigm. *Phys. Rev. B* **2008**, *77*, 184104.
(30) Goldschmidt, V. M. Die Gesetze Der Krystallochemie. *Naturwissenschaften* **1926**, *14* (21), 477–485. https://doi.org/10.1007/BF01507527.
(31) Shimakawa, Y.; Kubo, Y.; Nakagawa, Y.; Kamiyama, T.; Asano, H.; Izumi, F. Crystal Structures and Ferroelectric Properties of $SrBi_2Ta_2O_9$and $Sr_{0.8}Bi_{2.2}Ta_2O_9$. *Appl. Phys. Lett.* **1999**, *74* (13), 1904–1906. https://doi.org/10.1063/1.123708.
(32) Suárez, D. Y.; Reaney, I. M.; Lee, W. E. Relation between Tolerance Factor and T_c in Aurivillius Compounds. *J. Mater. Res.* **2001**, *16* (11), 3139–3149. https://doi.org/10.1557/JMR.2001.0433.
(33) Hervoches, C. H.; Snedden, A.; Riggs, R.; Kilcoyne, S. H.; Manuel, P.; Lightfoot, P. Structural Behavior of the Four-Layer Aurivillius-Phase Ferroelectrics $SrBi_4Ti_4O_{15}$ and $Bi_5Ti_3FeO_{15}$. *J. Solid State Chem.* **2002**, *164* (2), 280–291. https://doi.org/10.1006/jssc.2001.9473.
(34) Nistor, L.; Tendeloo, G. van; Amelinckx, S. The Paraelectric-Ferroelectric Phase Transition of $Bi_4Ti_3O_{12}$ Studied by Electron Microscopy. *Phase Transit.* **1996**, *59* (1–3), 135–153. https://doi.org/10.1080/01411599608220041.
(35) Hirata, T.; Yokokawa, T. Variable-Temperature X-Ray Diffraction of the Ferroelectric Transition in $Bi_4Ti_3O_{12}$. *Solid State Commun.* **1997**, *104* (11), 673–677. https://doi.org/10.1016/S0038-1098(97)00401-8.
(36) Hervoches, C. H.; Lightfoot, P. A Variable-Temperature Powder Neutron Diffraction Study of Ferroelectric $Bi_4Ti_3O_{12}$. *Chem. Mater.* **1999**, *11* (11), 3359–3364. https://doi.org/10.1021/cm991090d.
(37) Kubel, F.; Schmid, H. X-Ray Room Temperature Structure from Single Crystal Data, Powder Diffraction Measurements and Optical Studies of the Aurivillius Phase $Bi_5(Ti_3Fe)O_{15}$. *Ferroelectrics* **1992**, *129* (1), 101–112. https://doi.org/10.1080/00150199208016980.
(38) García-Guaderrama, M.; Fuentes-Montero, L.; Rodriguez, A.; Fuentes, L. Structural Characterization of $Bi_6Ti_3Fe_2O_{18}$ Obtained by Molten Salt Synthesis. *Integr. Ferroelectr.* **2006**, *83* (1), 41–47. https://doi.org/10.1080/10584580600949063.
